# Oxygen delivery, carbon dioxide removal, energy transfer to lungs and pulmonary hypertension behavior during venous-venous extracorporeal membrane oxygenation support: a mathematical modeling approach

**DOI:** 10.5935/0103-507X.20190018

**Published:** 2019

**Authors:** Bruno Adler Maccagnan Pinheiro Besen, Thiago Gomes Romano, Rogerio Zigaib, Pedro Vitale Mendes, Lívia Maria Garcia Melro, Marcelo Park

**Affiliations:** 1 Unidade de Terapia Intensiva, Disciplina de Emergências Clínicas; Departamento de Clínica Médica; Hospital das Clínicas, Faculdade de Medicina, Universidade de São Paulo - São Paulo (SP), Brasil.; 2 Unidade de Terapia Intensiva, Hospital da Luz - São Paulo (SP), Brasil.; 3 Unidade de Terapia Intensiva Oncológica, Hospital São Luiz Rede D'Or - São Paulo (SP), Brasil.; 4 Departamento de Nefrologia, Faculdade de Medicina do ABC - Santo André (SP), Brasil.; 5 Unidade de Terapia Intensiva, AC Camargo Cancer Center - São Paulo (SP), Brasil.; 6 Unidade de Terapia Intensiva, Hospital TotalCor - São Paulo (SP), Brasil.

**Keywords:** Respiratory failure, Acute Respiratory Distress Syndrome, Mechanical ventilation, Extracorporeal membrane oxygenation, Intensive care unit, Mathematical model, Insuficiência respiratória, Síndrome da angústia respiratória aguda, Ventilação mecânica, Oxigenação por membrana extracorpórea, Unidade de terapia intensiva, Modelo matemático

## Abstract

**Objective:**

To describe (1) the energy transfer from the ventilator to the lungs, (2) the match between venous-venous extracorporeal membrane oxygenation (ECMO) oxygen transfer and patient oxygen consumption (VO_2_), (3) carbon dioxide removal with ECMO, and (4) the potential effect of systemic venous oxygenation on pulmonary artery pressure.

**Methods:**

Mathematical modeling approach with hypothetical scenarios using computer simulation.

**Results:**

The transition from protective ventilation to ultraprotective ventilation in a patient with severe acute respiratory distress syndrome and a static respiratory compliance of 20mL/cm H_2_O reduced the energy transfer from the ventilator to the lungs from 35.3 to 2.6 joules/minute. A hypothetical patient, hyperdynamic and slightly anemic with VO_2_ = 200mL/minute, can reach an arterial oxygen saturation of 80%, while maintaining the match between the oxygen transfer by ECMO and the VO_2_ of the patient. Carbon dioxide is easily removed, and normal PaCO_2_ is easily reached. Venous blood oxygenation through the ECMO circuit may drive the PO_2_ stimulus of pulmonary hypoxic vasoconstriction to normal values.

**Conclusion:**

Ultraprotective ventilation largely reduces the energy transfer from the ventilator to the lungs. Severe hypoxemia on venous-venous-ECMO support may occur despite the matching between the oxygen transfer by ECMO and the VO_2_ of the patient. The normal range of PaCO_2_ is easy to reach. Venous-venous-ECMO support potentially relieves hypoxic pulmonary vasoconstriction.

## Abstract (pt)

**Objetivo:**

Descrever a transferência de energia do ventilador mecânico para os pulmões; o acoplamento entre a transferência de oxigênio por oxigenação por membrana extracorpórea venovenosa (ECMO-VV) e o consumo de oxigênio do paciente; a remoção de dióxido de carbono com ECMO; e o efeito potencial da oxigenação venosa sistêmica na pressão arterial pulmonar.

**Métodos:**

Modelo matemático com cenários hipotéticos e utilização de simulações matemáticas por computador.

**Resultados:**

A transição de ventilação protetora para ventilação ultraprotetora em um paciente com síndrome da angústia respiratória aguda grave e complacência respiratória estática de 20mL/cmH_2_O reduziu a transferência de energia do ventilador para os pulmões de 35,3 para 2,6 joules por minuto. Em um paciente hipotético, hiperdinâmico e ligeiramente anêmico com consumo de oxigênio de 200mL/minuto, é possível atingir saturação arterial de oxigênio de 80%, ao mesmo tempo em que se mantém o equilíbrio entre a transferência de oxigênio pela ECMO e o consumo de oxigênio do paciente. O dióxido de carbono é facilmente removido e a pressão parcial de dióxido de carbono normal é facilmente obtida. A oxigenação do sangue venoso, por meio do circuito da ECMO, pode direcionar o estímulo da pressão parcial de oxigênio na vasoconstrição pulmonar por hipóxia para valores normais.

**Conclusão:**

A ventilação ultraprotetora reduz amplamente a transferência de energia do ventilador para os pulmões. A hipoxemia grave no suporte com ECMO-VV pode ocorrer, a despeito do acoplamento entre a transferência de oxigênio, por meio da ECMO, e o consumo de oxigênio do paciente. A faixa normal de pressão parcial de dióxido de carbono é fácil de atingir. O suporte com ECMO-VV potencialmente alivia a vasoconstrição pulmonar hipóxica.

## INTRODUCTION

Venous-venous extracorporeal membrane oxygenation (VV-ECMO) has been successfully used to rescue acute respiratory distress syndrome (ARDS) patients with severe hypoxemia refractory to first line maneuvers.^(1-4)^ Mechanistically, respiratory ECMO support allows ultraprotective ventilation,^(5-7)^ avoiding additional unintended ventilator-associated lung injury^(8)^ and its systemic consequences.^(9)^

At the beginning of the VV-ECMO support, ultraprotective ventilation leads to a reduction in the mean airway pressure, while the ECMO circuit triggers a systemic inflammatory reaction.^(10)^ These two phenomena result in a dramatic decrease in the function of the native lungs, a situation clinically expressed as a quiet thorax, severe hypoxemia, and worsened pulmonary infiltrates ("white-out" phenomenon).^(11)^ Notably, the limited surface area of oxygenators (1.2 - 1.8m^2^) is sufficient for efficient blood decarboxylation;^(12-15)^ however, the ECMO oxygenation capacity is not as efficient.

The reduced native lung residual function associated with the limited capacity of ECMO oxygenation usually results in lungs well protected from the mechanical ventilator, with a patients' partial pressure of carbon dioxide (PaCO_2_) values close to normal and an arterial saturation of oxygen (SatO_2_) as low as 70%.^(6,16)^ Despite hypoxemia, VV-ECMO supported patients have had good outcomes,^(6,17,18)^ although the concept of permissive hypoxemia is still a matter of debate.

Our primary aim in this manuscript is to mathematically describe this scenario by analyzing the energy transfer from the ventilator to the lungs during protective and ultraprotective ventilation; the match between the oxygen transfer from ECMO to the oxygen consumption of the patient (despite hypoxemia); the efficiency of oxygenator blood decarboxylation; and the potential of VV-ECMO to reduce the pulmonary arterial pressure secondary to hypoxemia.

## METHODS

In this manuscript, four dimensions regarding the physiology of patients with severe respiratory failure under extracorporeal respiratory support will be explored: (1) the energy transfer from the ventilator to the lungs during normal, protective and ultraprotective ventilatory modes, through the measurement of the mechanical power (energy expressed in joules/minute); (2) the systemic oxygen supply during very low arterial oxygenation; (3) carbon dioxide removal; (4) the resulting oxygen partial pressure determinant of hypoxic pulmonary vasoconstriction stimulus (PO_2_-stimulus).

The principles of mathematical modeling, given its complexity, are shown in the supplementary material, including the formulas, the mathematical terms used for each dimension and the loops and iterations of the model (Figures 1S - 20S - Supplementary material). To build this model, we used physiological rationale based on standard formulas for the mechanical power, blood oxygen content and blood carbon dioxide content. Figure 2S (Supplementary material) shows the blood flows, and the oxygen and carbon dioxide content in blood used to perform the simulations. To assess the model consistency and stability, some simulations were performed (Figures 8S - 14S, 16S - 20S - Supplementary material).

After considering the model adequate for simulations, we chose a prototypical clinical scenario of a patient with severe ARDS. First, we considered a 36-year-old female patient with refractory hypoxemia due to Influenza A H1N1 pneumonia. During the second day of mechanical ventilation, the patient presented with a partial pressure of oxygen (PaO_2_) = 36mmHg, SatO_2_ = 64%, partial pressure of carbon dioxide (PaCO_2_) = 62mmHg, and pH = 7.24 despite prone positioning and alveolar recruitment maneuvers. Protective mechanical ventilation was applied (positive end-expiratory pressure - PEEP = 18cmH_2_O, fraction of inspired oxygen - FiO_2_ = 1; inspiratory time - T_insp_ = 0.6 s; I:E = 1:2; and tidal volume - Vt = 6mL/kg of ideal body weight - 360mL -, resulting in a plateau pressure - P_plat_ = 38cmH_2_O, static respiratory compliance - C_st_ = 18mL/cmH_2_O) with a respiratory rate = 35 breaths per minute (bpm). Given this scenario, the patient was started on VV-ECMO support.

After the first adjustments of the ECMO, the patient had an ECMO blood flow = 4900mL/minute, and a sweep gas flow = 3L/minute (FiO_2_ = 1), using heparin. Mechanical ventilation was transitioned to ultraprotective ventilation using the pressure control mode (PCV) with PEEP = 14cmH_2_O, driving pressure = 10cmH_2_O, and T_insp_ = 1 s, resulting in a Vt of 14mL (C_st_ = 14mL/cmH_2_O), RR = 10bpm, and FiO_2_ = 0.3. The arterial blood gas analysis (ABG) shows a pH = 7.38, PaO_2_ = 52mmHg, PaCO_2_ = 35mmHg, and SatO_2_ = 84%. The cardiac output is 10L/minute and hemoglobin = 10g/dL.

All mathematical modeling and simulations were performed in C language using R free source software.^(19)^

## RESULTS

The results of the mathematical modeling are shown according to the dimension analyzed.

### Energy transfer from ventilator to the lungs

**Figure 1:**
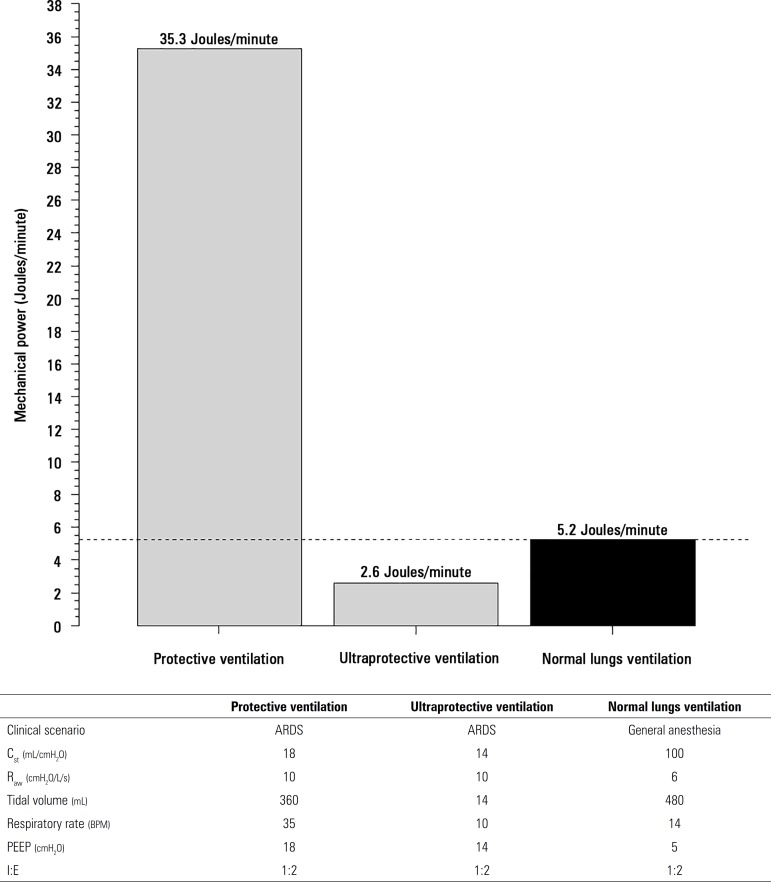
Mechanical power expressing the energy load per minute transferred from the mechanical ventilator to the lungs. The clinical scenario of the ARDS patient is described in the text. Normal lung ventilation expresses the mechanical power of a healthy patient under general anesthesia for elective surgery. R_aw_ - airway resistance; C_st_ - static respiratory compliance; I:E - I/E time ratio.

Figure 1 shows the energy transfer from the ventilator to the lungs before ECMO installation during protective ventilation, which was as high as 35.3 joules/min. By contrast, after the initiation of ultraprotective ventilation, it reached values as low as 2.6 joules/min, comparable to a healthy patient with normal lungs undergoing standard intraoperative ventilation (5.2 joules/min). The airway resistance (R_aw_) for the calculation was 10cmH_2_O/L/second.

Increasing the PEEP and respiratory rate led to a linear increase in the mechanical power (Figures 21S and 22S - Supplementary material). Increases in driving pressure (DP) also led to a quasi-linear increase in the mechanical power (Figure 23S - Supplementary material). The effect of increasing the inspiratory-to-expiratory time ratio (I:E) on mechanical power was small, except when the inspiratory time was greater than 4 s (Figure 24S - Supplementary material). The driving pressure effect on the mechanical power had a weak association with increasing levels of PEEP but had a more important association with increasing respiratory rates (Figure 25S - Supplementary material). Figure 26S (Supplementary material) shows the potential effect of airway pressure release ventilation (APRV) on the mechanical power, which can be a strategy to increase mean airway pressure levels while maintaining the energy transfer to the lungs at lower values.

### Arterial oxygenation and total amount of oxygen transfer

**Figure 2:**
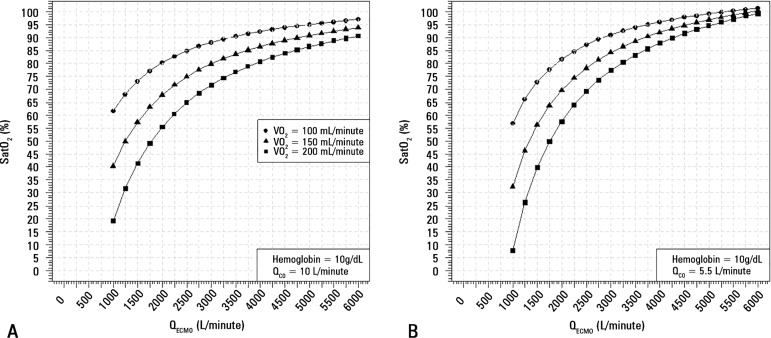
Arterial oxygen saturation with increasing extracorporeal membrane oxygenation blood flow, for different oxygen consumption levels and a shunt fraction fixed at 95%. (A) shows this relation with cardiac output of 10L/minute and (B) shows this relation with cardiac output of 5.5L/minute. SatO_2_ - arterial oxygen saturation; VO_2_ - oxygen consumption; Q_CO_ - cardiac output; Q_ECMO_ - extracorporeal membrane oxygenation blood flow.

Figure 2 demonstrates that increasing VV-ECMO blood flow leads to better arterial oxygenation and total amount of oxygen transfer. In this figure, the pulmonary shunt was considered to be 95%. The prototypical patient is represented as having a VO_2_ of 200mL/minute (Figure 2 - Panel A), which leads, despite low oxygen saturation levels, to an adequate oxygen supply for the patient's needs.

For a given patient with high cardiac output (10L/min), a higher hemoglobin level (10g/dL compared to 7g/dL) leads to a higher SatO_2_ (Figures 27S and 28S - Supplementary material). Furthermore, higher values of hemoglobin (14g/dL) do not seem to provide a meaningful increase in oxygenation. At a lower cardiac output (5.5L/min), even for a hypothetical patient with low hemoglobin levels (7g/dL), reasonable oxygen saturation may be achieved by increasing the ECMO blood flow, although a higher hemoglobin level will also lead to better SatO_2_ in this context (Figure 29S - Supplementary material). In the context of a shunt fraction of 100% and high cardiac output, one can observe that it is hard to achieve reasonable SatO_2_ with low hemoglobin values (Figure 30S - Supplementary material).

### Arterial carbon dioxide and total amount of carbon dioxide transfer

**Figure 3:**
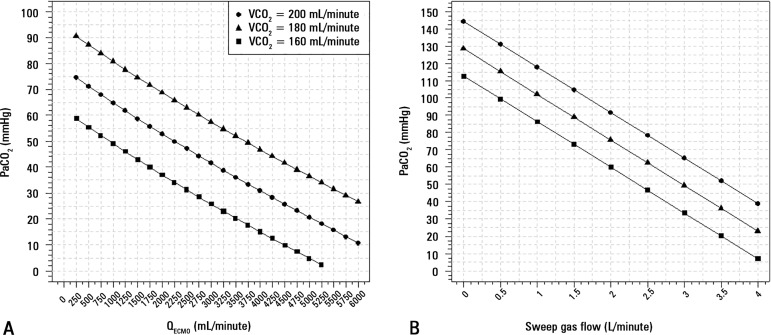
Variation of partial pressure of carbon dioxide with increasing extracorporeal membrane oxygenation blood flow with a sweep gas flow fixed in 3.5L/minute (A), and sweep gas flow with extracorporeal membrane oxygenation blood flow = 3500mL/minute (B) for three different levels of carbon dioxide production. PaCO_2_ - partial pressure of carbon dioxide; VCO_2_ - carbon dioxide production; Q_ECMO_ - extracorporeal membrane oxygenation blood flow.

Figure 3 shows the effect of increasing ECMO support, either through increasing ECMO blood flow or sweep gas flow, on decreasing the arterial carbon dioxide partial pressure. Our patient is represented as having a VCO_2_ of approximately 160mL/minute. Higher cardiac outputs do not lead to worsened CO_2_ transfer, while lower hemoglobin levels may lead to a small reduction in CO_2_ transfer (Figure 31S - Supplementary material).

### Oxygen partial pressure responsible for pulmonary vasoconstriction inhibition (P_stimulus_ O_2_)

**Figure 4:**
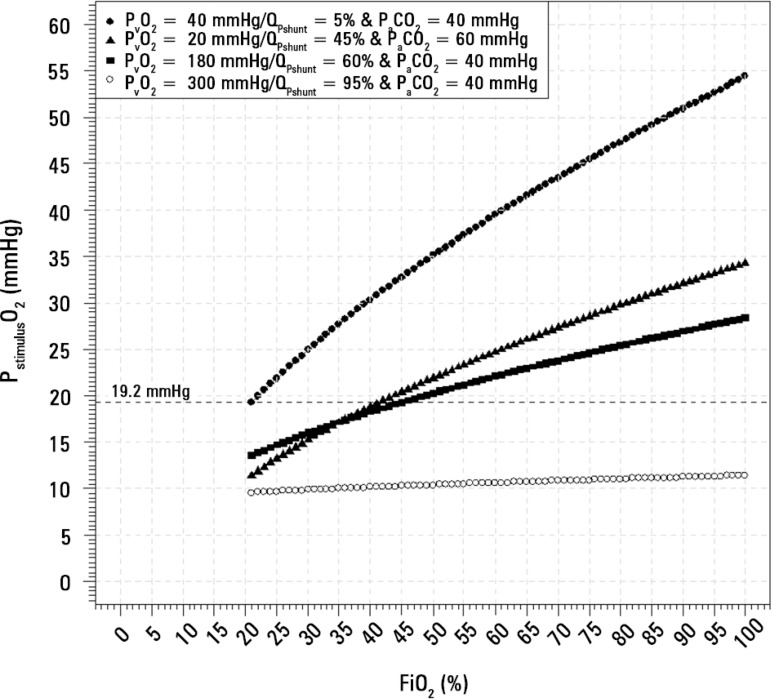
Oxygen partial pressure responsible for the hypoxic pulmonary vasoconstriction inhibition in four different clinical scenarios. The dotted line at P_stimulus_O_2_ of 19.2mmHg represents the partial pressure during breathing of a healthy person. The other clinical scenarios reproduce the mechanical ventilation in a severe ARDS patient with hypercapnia, a pulmonary shunt fraction of 45%, and P_v_O_2_ = 20mmHg (closed triangle); the closed squares represent the same patient after ECMO initiation, when the pulmonary shunt is increased to 60% (whitening-up phenomenon) and the P_v_O_2_ is increased to 180mmHg; the open circles represent the same patient with a pulmonary shunt of 95% and a P_v_O_2_ of 300mmHg. P_v_O_2_ - partial pressure of venous oxygen; Q_Pshunt_ - pulmonary shunt blood flow fraction; P_stimulus_O_2_ - hypoxic pulmonary vasoconstriction stimulus; FiO_2_ - fraction of inspired oxygen.

Figure 4 demonstrates the effect of ECMO support on P_stimulus_O_2_. Placing the patient on ECMO may initially increase hypoxic vasoconstriction, but this may be corrected with increased venous oxygen partial pressure (PvO_2_) levels through ECMO. However, if the shunt fraction increases excessively (Figure 4 - open circles), one may not be able to release the hypoxic vasoconstriction effect on the pulmonary circulation.

Figure 32S (Supplementary material) demonstrates the more deleterious effect of progressively higher shunt fractions on P_stimulus_O_2_ when compared to lower PvO_2_ levels, according to the FiO_2_ levels.

## DISCUSSION

This case-based mathematical model shows that ultraprotective ventilation largely reduces the energy transfer from the ventilator to the lungs. The price for this initial lung rest is hypercapnia and hypoxemia. The PaCO_2_ is easily compensated through VV-ECMO support. The oxygenation, however, can reach critically low levels, which are, nevertheless, adequate to match the patient's oxygen consumption. Predominant arterial pulmonary oxygenation from VV-ECMO support potentially normalizes the oxygenation trigger for hypoxic pulmonary artery vasoconstriction and hypertension, although allowing the patient to reach shunt fractions that are too high may blunt the effect of ECMO on hypoxic pulmonary vasoconstriction. Although the findings of this mathematical model are not entirely novel, the combined description of these physiological alterations may help physicians make decisions at the bedside regarding transfusion, ventilator settings, ECMO settings and initial ECMO configuration, due to the lack of data on ECMO-treated patients that would enable these decisions.

The ventilator-associated lung injury depends on the mechanical distention of the respiratory system resulting in lung stretching.^(8)^ This pulmonary mechanical deformation depends on energy transfer from the mechanical ventilator to the lungs that can be accurately calculated through the mechanical power.^(20)^ The dynamic energy transfer, i.e., the energy transferred in each respiratory cycle, is the main factor responsible for parenchymal lung damage,^(21,22)^ and lung rest with some applied positive end-expiratory pressure (PEEP) is associated with less lung damage.^(21)^ The mechanical power reduction shown in this modeling is potentially one of the mechanisms of better outcomes of patients undergoing ultraprotective ventilation during respiratory ECMO support.^(23,24)^ This large reduction of mechanical power encourages the practice of UP ventilation at the start of ECMO support. We argue that, as with standard protective ventilation, after 12 - 72 hours of UP ventilation, maintaining this strategy is not necessarily beneficial^(25,26)^ and may lead to unnecessary heavy sedation and/or deleterious prolonged neuromuscular blockade; therefore, further investigations are needed.

After long-term evaluation, severe hypoxemia is associated with a higher occurrence of neurocognitive deficits,^(27,28)^ despite the lack of association between hypoxemia and mortality in adult ARDS patients.^(29)^ During the first hours of VV-ECMO support, severe hypoxemia is common;^(6,16)^ however, it is not associated with neurocognitive deficits.^(18)^ One can suppose that hypoxemia without concomitant respiratory acidosis, as well as the reduction of sedative needs, can contribute to the divergent neurocognitive findings.^(6,30)^ The higher the hemoglobin level, the higher the oxygen delivery.^(31)^ Therefore, many groups use hemoglobin levels of at least 10g/dL,^(6)^ and up to 14g/dL.^(1,5)^ In fact, our model shows that the higher the hemoglobin level, the higher the arterial oxygen saturation (higher the oxygen content) for the same oxygen consumption. Nevertheless, low hemoglobin levels allow the equilibrium between ECMO oxygen transfer and the patient's oxygen consumption at lower oxygen saturation levels. Therefore, severe hypoxemia during VV-ECMO support does not necessarily equate to oxygen delivery to consumption mismatch, and other factors, such as normalization of lactate level and metabolic acidosis, should help the intensivist to interpret the clinical scenario of severe hypoxemia.^(18)^ Another issue leading to poor oxygenation may be high cardiac output, as already described, due to the high portion of cava blood flow that bypasses the extracorporeal device.^(16)^ In this regard, lower hemoglobin values may further reduce the oxygen transfer to the patient.

Finally, lung protective mechanical ventilation, which is associated with concomitant hypercapnia and hypoxemia, may lead to an incidence of 51% of acute *cor pulmonale*.^(32)^ In this context, hemodynamic instability may lead to veno-arterial (VA)-ECMO configuration initiation. However, as hypoxemia (both alveolar and venous) may have an important role in hypoxic pulmonary vasoconstriction and secondary pulmonary hypertension, the large oxygenation of cava venous return during VV-ECMO can alleviate the *cor pulmonale*, precluding VA-ECMO use. In fact, the pulmonary pressure reduction and improvement of right ventricle function after VV-ECMO initiation is notorious in ARDS patients.^(33)^ In our results, the elevation of P_v_O_2_ during the VV-ECMO support can reach a PO_2stimulus_ similar to physiological conditions.

This study has several limitations. First, it was designed to help understand the VV-ECMO support at bedside, but it should not be blindly used to guide the support primarily because, as a mathematical model, it does not account for many immeasurable and undetectable physiological factors. Second, many predictable variables may not respond in the way we described after temperature, pH, P_a_CO_2_ or other physiological variations are considered. Third, there are no biological data to confirm our findings in this report, although many reports in the literature have described the phenomena we tried to depict in this mathematical model.

## CONCLUSION

This model shows that venous-venous extracorporeal membrane oxygenation support facilitates ultraprotective ventilation, which is associated with an impressive reduction in energy transfer from ventilator to the lungs. Ultraprotective ventilation during the first moments of venous-venous extracorporeal membrane oxygenation support may be associated with severe hypoxemia, although the match between extracorporeal membrane oxygenation oxygen transfer and the patient's oxygen consumption is maintained. Despite severe hypoxemia, a normal range of P_a_CO_2_ is easily attainable. The large elevation of venous oxygen partial pressure during venous-venous extracorporeal membrane oxygenation potentially relieves hypoxic pulmonary vasoconstriction, reducing pulmonary pressure and improving acute *cor pulmonale*.
