# Supplementary material for: Oxygen delivery, carbon dioxide removal, energy transfer to lungs and pulmonary hypertension behavior during venous-venous extracorporeal membrane oxygenation support: a mathematical modeling approach
Source: Rev Bras Ter Intensiva. 2019 Apr-Jun;31(2):113–21. doi: 10.5935/0103-507X.20190018 (PMC6649222; doi:10.5935/0103-507X.20190018)
Supplement: Supplementary file 1 [file rbti-31-02-0113-suppl1.pdf]

Oxygen delivery, carbon dioxide removal, energy transfer to lungs and pulmonary hypertension behavior during venous-venous extracorporeal membrane oxygenation support: a mathematical modeling approach

Oferta de oxigênio, remoção de dióxido de carbono, transferência de energia aos pulmões e comportamento da hipertensão pulmonar durante o suporte com oxigenação por membrana extracorpórea: um modelo matemático

Bruno Adler Maccagnan Pinheiro Besen[1,2], Thiago Gomes Romano[3,4], Rogerio Zigaib[1,5], Pedro Vitale Mendes[1,3], Lívia Maria Garcia Melro[1,6], Marcelo Park[1]

INDEX

1. METHODS 3

1.1. Energy transfer from ventilator to the lungs........ 3

1.2. Arterial oxygenation and total amount of oxygen transfer 6

1.2.1. Standard formulas used........ 8

1.2.2. Creating mathematical terms of the oxygen modeling........ 8

1.2.3. Looping and iteractions to reach the equilibrium........ 15

1.3. Arterial carbon dioxide and total amount of carbon dioxide transfer 23

1.3.1. Standard formulas used........ 23

1.3.2. Creating mathematical terms of the carbon dioxide modeling 23

1.3.3. Looping and iteractions to reach the equilibrium........ 27

1.4. Oxygen partial pressure responsible for pulmonary vasoconstriction inhibition ($P_{stimulus}O_2$). 36

2. RESULTS 36

2.1. Energy transfer from ventilator to the lungs........ 36

2.2. Arterial oxygenation and total amount of oxygen transfer 42

2.3. Arterial carbon dioxide and total amount of carbon dioxide transfer 44

2.4. Oxygen partial pressure responsible for pulmonary vasoconstriction inhibition ($P_{stimulus}O_2$). 45

3. REFERENCES........ 46

4. APENDIX 48

4.1. Script for $SatO_2$ looping........ 48

4.2. Script for P_aCO_2 looping........ 49

1. METHODS

In this supplementary material, we describe the principles of the mathematical modeling.

1.1. Energy transfer from the ventilator to the lungs

The basic principle of mechanical ventilation relies on active pulmonary inflation through the energy transfer from the mechanical ventilator to the lungs. In acute respiratory distress syndrome (ARDS) patients, the higher the pulmonary compliance and the lower the airway resistance, the lower will be ventilator-to-lungs energy transfer, and a high proportion of this low amount of energy transfer will be dissipated generating tidal volume (Vt) (Figure 1S). Likewise, low pulmonary compliance with low airway resistance will be associated with a high amount of the ventilator-lungs energy transfer dissipated in alveoli overdistension and cyclic opening of respiratory units, a process determinant of the physical concept of lungs strain, in which the higher the strain, the more the lungs expand relatively to initial expiratory lung volumes.[1,2] The strain is partitioned in static strain, as generated by positive end-expiratory pressure (PEEP), and dynamic strain, as generated by Vt. The dynamic strain results in deformation of the lung structure, triggers inflammation, edema and hemorrhage formation. Furthermore, a high static strain is associated with slight inter-alveolus septum rupture and interstitial emphysema development.[2,3]

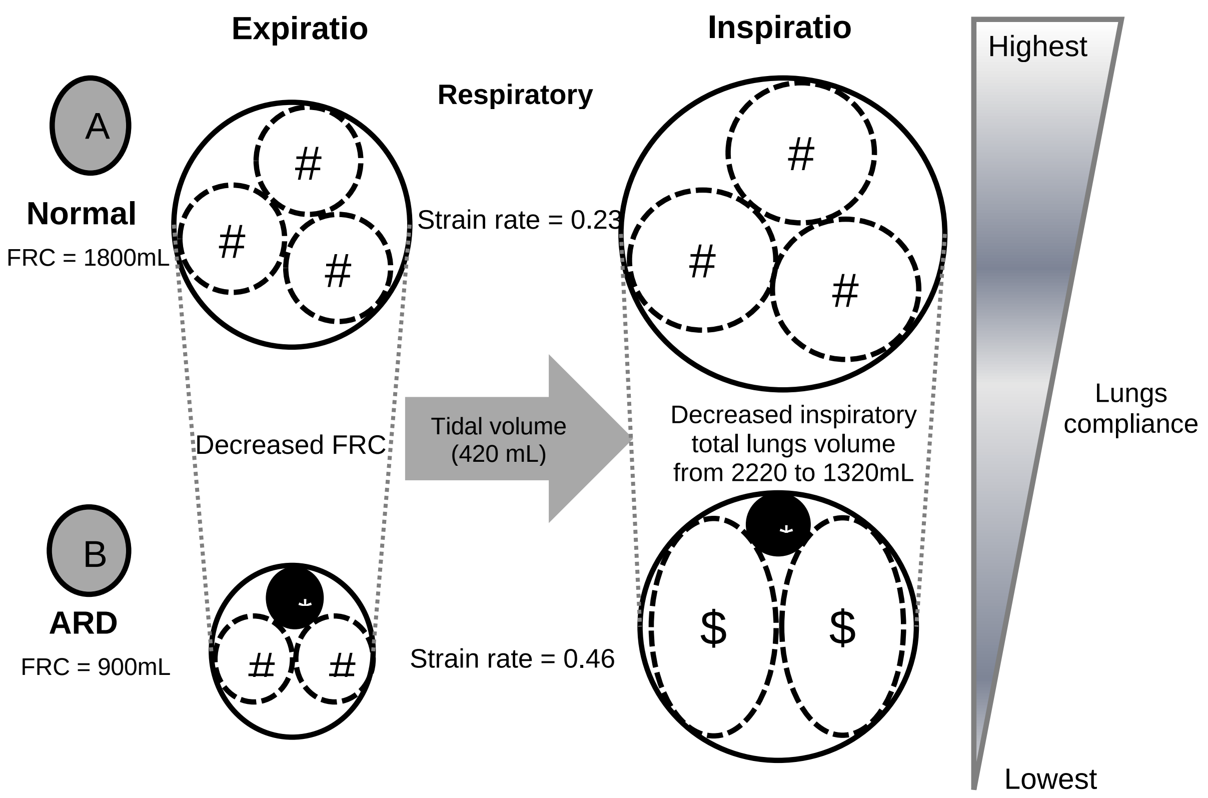

Figure 1S - Exploring the strain concept. Defining strain rate as tidal volume/ functional residual capacity ratio, in **Panel A)** (Normal lungs condition), the higher functional residual capacity promotes a lower strain rate than **Panel B)** (acute respiratory distress syndrome condition), considering the tidal volume is the same. In acute respiratory distress syndrome, the collapsed alveolus causes a lower pulmonary compliance than in normal condition; therefore the energy spent generating tidal volume in normal condition is also spent in mechanical alveoli hyperdistention (higher strain rate) as presented in acute respiratory distress syndrome condition. FRC – functional residual capacity; ARDS - acute respiratory distress syndrome. # Normally aerated alveoli; * collapsed alveoli; $ hyperdistended alveoli.

In order to quantify the amount of energy transferred from the ventilator to the lung, the mechanical power concept was used.[4] The mechanical power is derived from the equation of motion, and then computes the most important energy load components (Elastic and resistive, adding PEEP to the equation and neglecting inertial forces) of each respiratory cycle.[5] To quantify the total energy load transference per minute, the obtained value is multiplied by the respiratory rate. The calculation of energy load transferred from the mechanical ventilator to the lungs per minute is mathematically expressed as following:[4]

$$Power_{rs} = 0.098 * RR * \{▲V^2 * [½ * EL_{rs} + RR * [(1 + I:E) / (60 * I:E)] * R_{aw}] + ▲V * PEEP\}$$

Where:

$Power_{rs}$ - energy transferred to the lungs (Joules/minute)

RR - respiratory rate (Breaths/minute)

▲V - tidal volume (mL)

EL_{rs} - respiratory system elastance (cmH_2O/L)

I:E - inspiratory time / expiratory time ratio

R_{aw} - airway resistance (cmH_2O/L/second)

PEEP - positive end-expiratory pressure (cmH_2O)

The calculation of the mechanical power has a good correlation and a reasonable agreement with the tomographic measurement of the pulmonary energy load in mechanically ventilated patients.(4)

Some ventilatory settings are used during ultraprotective ventilation beside extracorporeal membrane oxygenation (ECMO) support, with different impact on mechanical power. Since pressure controlled modality (PCV) is frequently used in ECMO supported patients, the energy load related variables adjusted at bedside are: respiratory rate (RR), PEEP, I:E, and driving pressure. Ventilatory settings allowing lung rest are used in up to 66% of Extracorporeal Life-Support organization (ELSO) registered ECMO centers.(6) In this matter, the CESAR trial ventilatory strategy is a very attractive one.(7) Furthermore, this ventilatory strategy is widely used,(6,8,9) and also by our group.(10) This strategy includes the following ventilatory settings: PEEP = 10 - 15cmH_2O, RR = 10 - 15BPM, and a driving pressure of 5 - 15cmH_2O. There is an

association of higher PEEPs (10 - 15cmH_2O) and lower plateau or driving pressures with lower mortality.[8,11] The impact of I:E ratio on any outcome is not well established. Therefore, the marginal effect of each cited variable on the mechanical power was simulated in the supplementary results. As many ventilators offer the possibility of direct inspiratory time adjustment, the plot between inspiratory time normalized to a RR = 10BPM and mechanical power was also built.

In order to facilitate the understanding of PEEP interaction effect with driving pressure on the mechanical power, and RR interaction effect with driving pressure on the mechanical power, two graphs plotting driving pressure and mechanical power were built, one with different PEEP levels and other with different RRs.

The airway pressure release ventilation (APRV) is used in up to 11% of ELSO centers.[6] Our group has used the APRV in patients with alveolar hemorrhage or in patients with collapsed lung who is potentially recruitable. The APRV is also explored in the supplementary results section.

1.2. Arterial oxygenation and total amount of oxygen transfer

We did the mathematical modeling considering the venous-venous configuration with the drainage cannula introduced in the femoral vein, and the arterial cannula introduced in the jugular vein. The oxygenation, oxygen transfer, decarboxylation, and carbon dioxide transfer modeling were explored using the following model:

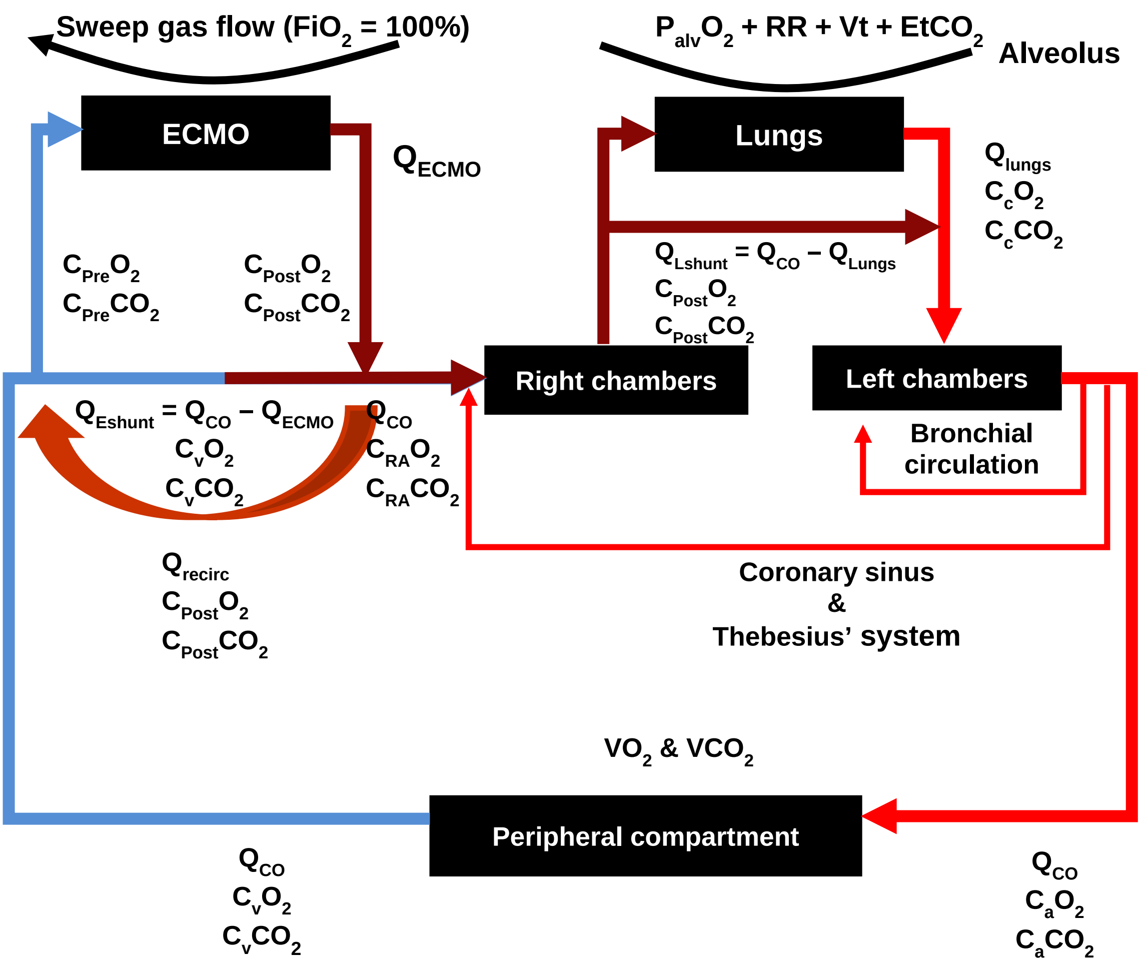

Figure 2S - Patient and extracorporeal membrane oxygenation coupling scheme of the mathematical modeling. In this figure, the blood flows, oxygen and carbon dioxide blood contents used in the mathematical modeling are depicted. FiO_2 - fraction of inspired oxygen; $P_{alv}O_2$ - oxygen alveolar partial pressure; RR - respiratory rate; Vt - tidal volume; ECMO - extracorporeal membrane oxygenation; $C_{pre}O_2$ - pre-extracorporeal membrane oxygenation oxygen blood content; $C_{post}O_2$ - post-extracorporeal membrane oxygenation oxygen blood content; $C_{pre}CO_2$ - pre-extracorporeal membrane oxygenation carbon dioxide blood content; $C_{post}CO_2$ - post-extracorporeal membrane oxygenation carbon dioxide blood content; Q_{ECMO} - extracorporeal membrane oxygenation blood flow; Q_{Eshunt} - extracorporeal membrane oxygenation parallel cava blood flow (cava blood flow without extracorporeal membrane oxygenation); Q_{CO} - cardiac output; C_vO_2 - venous oxygen blood content; $C_{RA}O_2$ - right atrium oxygen content; C_vCO_2 - venous carbon dioxide blood content; $C_{RA}CO_2$ - right atrium carbon dioxide content; Q_{recirc} - extracorporeal membrane oxygenation recirculation blood flow; $C_{post}CO_2$ - post-extracorporeal membrane oxygenation carbon dioxide blood content; Q_{Lungs} - pulmonary blood flow in matched aerated alveoli/capillaries; C_cO_2 - pulmonary alveolar capillary blood oxygen content; C_cCO_2 - pulmonary alveolar capillary carbon dioxide blood content; Q_{Lshunt} - pulmonary blood flow in shunt regions; VO_2 - peripheral compartment oxygen consumption; VCO_2 - peripheral compartment carbon dioxide production.

General nomenclature

VO_2 - peripheral compartment oxygen consumption.

VCO_2 - peripheral compartment carbon dioxide production.

$P_{alv}O_2$ - oxygen alveolar partial pressure.

$EtCO_2$ - end-tidal carbon dioxide partial pressure.

RR - respiratory rate

Vt - tidal volume

Blood Flows

Q_{CO} – cardiac output, Q_{ECMO} – ECMO blood flow, Q_{Eshunt} – ECMO parallel cava blood flow (cava blood flow without ECMO oxygenation), Q_{recirc} – ECMO recirculation blood flow, Q_{Lungs} – pulmonary blood flow in matched aerated alveoli / capillaries, Q_{Lshunt} – pulmonary blood flow in shunt regions.

Oxygen and carbon dioxide blood contents

C_aO_2 – arterial oxygen blood content, C_vO_2 - venous oxygen blood content, $C_{pre}O_2$ – pre-ECMO oxygen blood content, $C_{post}O_2$ - post-ECMO oxygen blood content, $C_{RA}O_2$ – right atrium oxygen content, and C_cO_2 – pulmonary alveolar capillary blood oxygen content.

C_aCO_2 – arterial carbon dioxide blood content, C_vCO_2 - venous carbon dioxide blood content, $C_{pre}CO_2$ – pre-ECMO carbon dioxide blood content, $C_{post}CO_2$ – post-ECMO carbon dioxide blood content, $C_{RA}CO_2$ – right atrium carbon dioxide content, and C_cCO_2 – pulmonary alveolar capillary carbon dioxide blood content.

1.2.1. Standard formulas used

- Blood local oxygen content (C_LO_2) = $PO_2 * 0.0031 + (1.34 * SatO_2 * Hb)$

- Local oxygen delivery (DO_{L2}) = $C_LO_2 * Q_L$

- $VO_2 = (C_a - C_v) * Q_{CO}$

- Oxygen transfer = $(C_{post}O_2 – C_{pre}O_2) * Q_{ECMO}$

- Pulmonary shunt (Q_S/Q_T) = $(C_cO_2 – C_aO_2) / (C_cO_2 – C_VO_2)$

1.2.2. Creating mathematical terms of the oxygen modeling

(1) Following the figure 2S, starting in the systemic venous compartment, computing the blood drained from the peripheral compartment:

From Fick’s equation:

$$S_vO_2 = SaO_2/100 – (VO_2 / (Q_{CO} \times Hb \times 1.36 \times 10))$$
and
$$C_vO_2 = S_vO_2 \times 1.36 \times Hb$$

Where Hb is the hemoglobin level in g/dL and Q_{CO} is the cardiac output only here is expressed in L/minute.

(2) ECMO recirculation term

Q_{recirc} has been mathematically described as a function of the Q_{ECMO}.[12-14] Using the optimized femoral-jugular configuration, with a Q_{ECMO} of 5000mL, it is expected a recirculation rate up to 20%.[12] During low Q_{ECMO} rate, the Q_{recirc} varies linearly according to the Q_{ECMO},[14] however, at higher Q_{ECMO} rate this relation of Q_{recirc} with Q_{ECMO} is non-linear (exponential function).[14]

Intuitively, the Q_{CO} is also a modulator of the recirculation once keeping Q_{ECMO} stable; the higher the Q_{CO}, the higher will be the right ventricular drainage of the cannulas region and smaller the recirculation rate due to the ventricular drainage of the arterial cannula blood output. In this way, an extremely low Q_{CO} is responsible for almost 100% of arterial cannula blood recirculation.[14] As a higher Q_{ECMO} is directly associated with a higher Q_{recirc}, and a higher Q_{CO} is associated with a lower Q_{recirc}, the equation was fitted using recirculation rate against the Q_{ECMO}/Q_{CO} ratio.

The recirculation rate equation was created as a single exponential function (basic equation: $f(x) = a * (1 - b^x)$), where f(x) is the recirculation rate and x is the Q_{ECMO}/Q_{CO} ratio. Fitting all the data exposed above resulted in the following equation:

$$\text{Recirculation rate (\%)} = 29.1321 * (1 - 0.2791^{Q_{ECMO}/Q_{CO}})$$

From here on, the Q_{CO} and Q_{ECMO} are expressed in mL/minute.

Plotting the fitted equation with Q_{ECMO} resulted in the following graphs:

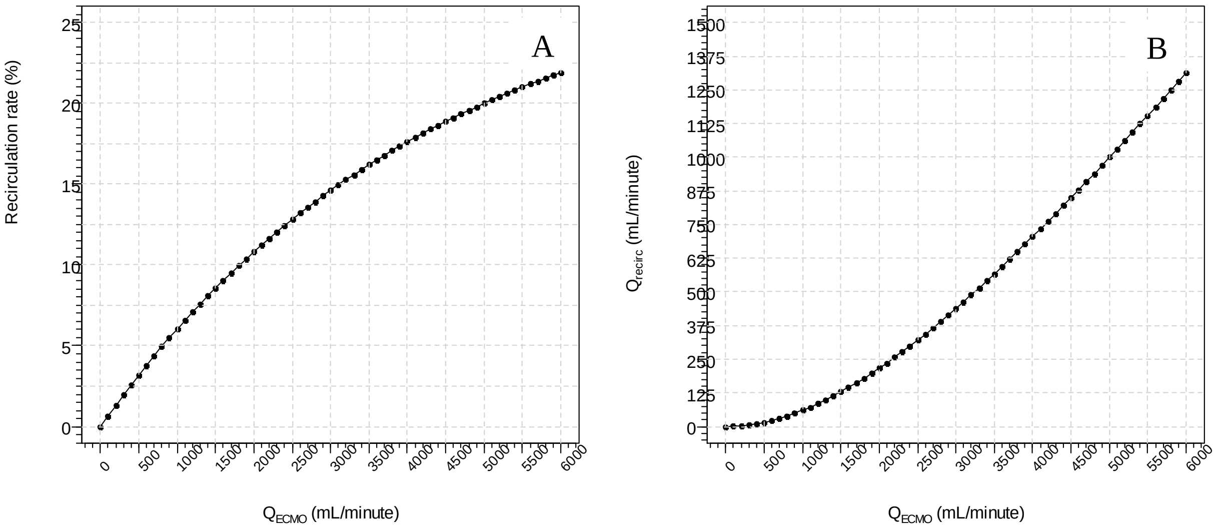

Figure 3S - Association of extracorporeal membrane oxygenation blood flow with recirculation rate (**Panel A**) and extracorporeal membrane oxygenation blood flow with extracorporeal membrane oxygenation recirculation blood flow (**Panel B**). In both simulations, cardiac output was fixed in 5500mL/minute.

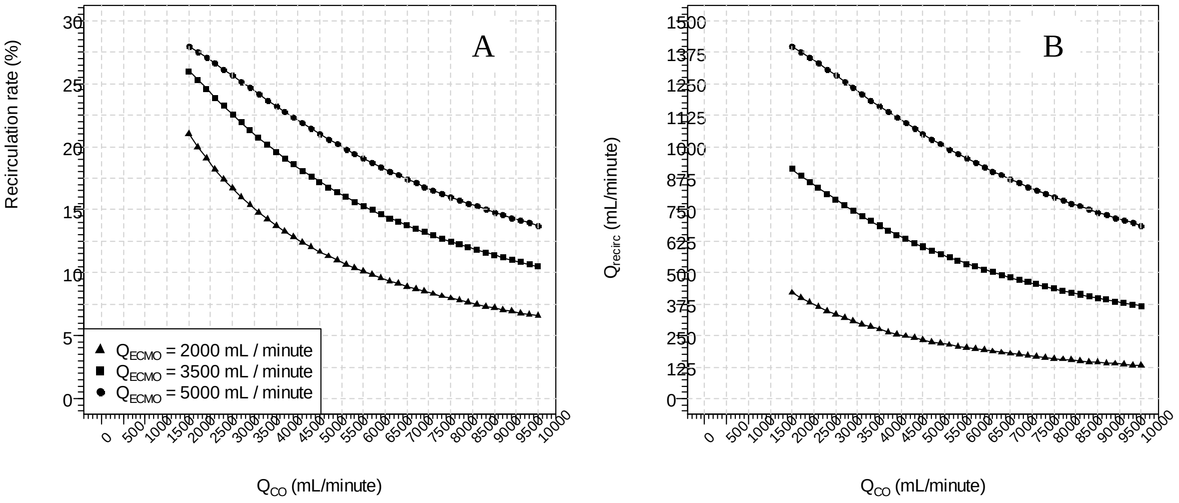

Figure 4S - Association of cardiac output with the recirculation rate (**Panel A**) and cardiac output with extracorporeal membrane oxygenation recirculation blood flow (**Panel B**). In both simulations, cardiac output varied from 2000 to 10,000mL/minute. Three different extracorporeal membrane oxygenation blood flows were simulated in order to demonstrate the interaction between both variables.

The recirculating blood flow carries $C_{Post}O_2$, therefore there will be not oxygenation of this amount of blood passing through the oxygenator. Thus, the effective Q_{ECMO} (the partition of Q_{ECMO} which will really be oxygenated) will be defined as:

$$Q_{ECMO}\text{ effective }(Q_{ECMOeff}) = Q_{ECMO} - Q_{recirc}$$

The $Q_{ECMOeff}$ plotted against the Q_{ECMO} (with Q_{CO} simulated in 2000, 5000, and 10,000mL) is shown below:

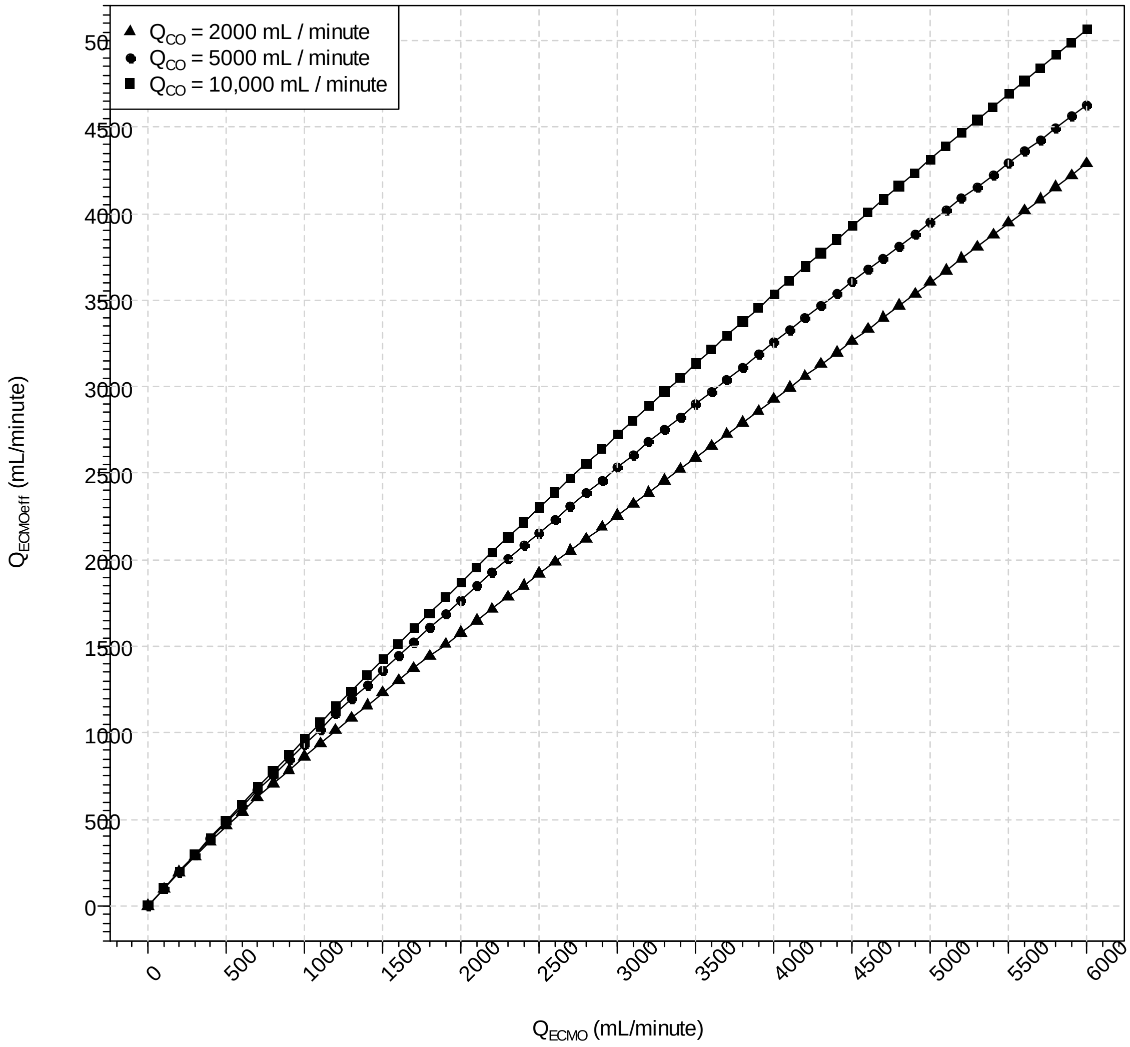

Figure 5S - Association of extracorporeal membrane oxygenation blood flow with the effective ECMO blood flow ($Q_{ECMOeff}$) in three different cardiac output.

(3) Post-oxygenator oxygen content ($C_{Post}O_2$)

The FiO_2 in the oxygenator sweep gas flow was considered 100%. Considering the oxygenator as efficient, the hemoglobin will ever be 100% saturated by oxygen. The $P_{Post}O_2$ at higher Q_{ECMO} is expected to be lower than $P_{Post}O_2$ at lower Q_{ECMO}.[15] The $P_{Post}O_2$ at Q_{ECMO} = 5500 - 6000mL/minute is expected to be ≥ 150mmHg,[16] and a Q_{ECMO} as low as 2000mL/minute the $P_{Post}O_2$ is expected to be around 400mmHg, with a non-linear $P_{Post}O_2$ decrement with progressive higher Q_{ECMO}.[12,15] In order to fit this non-

linear $P_{Post}O_2$ decrement, the formula used was based on an exponential decay f(x) = a + b * exp(1) ^ (-c * x), where f(x) is the $P_{Post}O_2$ and x is the Q_{ECMO}. The final plus 1 was inserted to avoid zero as denominator. The result of fitting the above data was:

$$P_{Post}O_2 = 130.5 + 319.5 * \exp(1) \wedge (-0.0005 * Q_{ECMO})$$

The $C_{Post}O_2$ was then calculated as:

$$C_{Post}O_2 = 0.0031 * P_{Post}O_2 * 1.36 * Hb$$

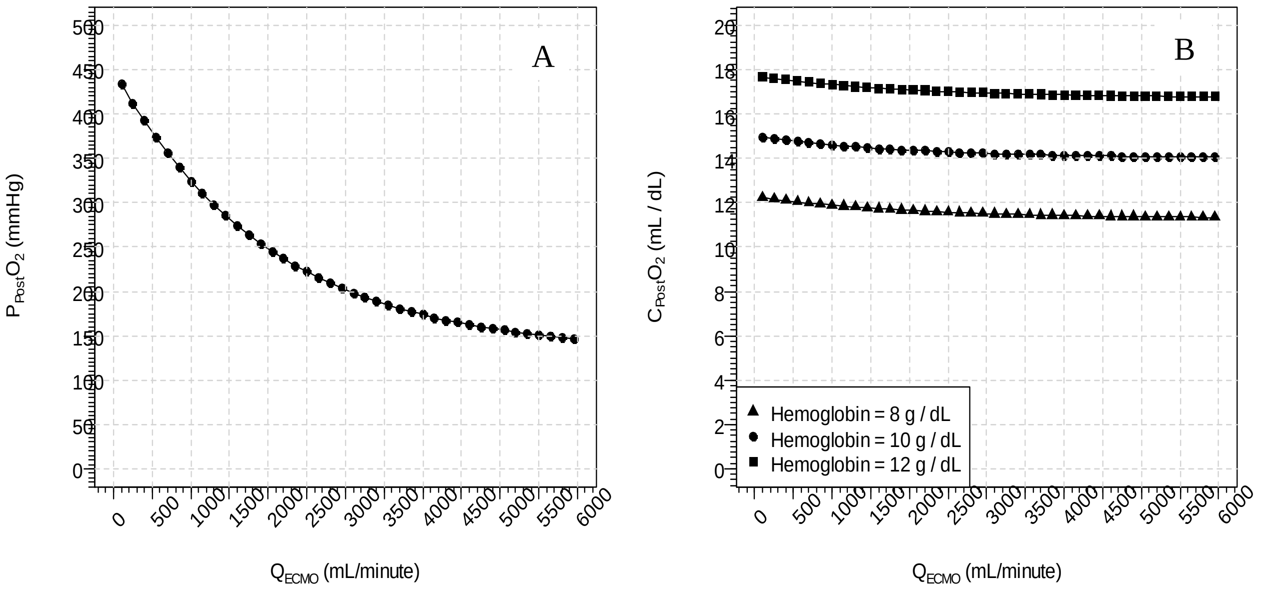

Figure 6S - Post-extracorporeal membrane oxygenation partial pressure of oxygen (**Panel A**) and post-extracorporeal membrane oxygenation oxygen blood content (**Panel B**) as a function of extracorporeal membrane oxygenation blood flow. The **Panel B** shows the association with three different levels of hemoglobin.

(4) The oxygen transfer in the oxygenator

The oxygen transfer is defined as following:[15]

$$\text{Oxygen transfer } (O_2 \text{ transfer}) = (C_{Post}O_2 - C_{Pre}O_2) * Q_{ECMOeff}$$

The $C_{Pre}O_2$ as a separated fraction from the recirculated blood is considered equal to C_VO_2. The simulation of O_2 transfer as function of Q_{ECMO} leads to the main result of this modeling, and is presented in the results section of the main manuscript.

(5) Right atrial oxygen venous content ($C_{RA}O_2$)

The coronary sinus blood flow (1mL/kg/minute) was considered negligible to an adult. The $C_{RA}O_2$ is the mean of the $C_{Post}O_2$ and C_VO_2 pondered according to the blood flows, that is:

$$Q_{Eshunt} = Q_{CO} – Q_{ECMOeff}$$

$$C_{RA}O_2 = [(C_{Post}O_2 * Q_{ECMOeff}) + C_VO_2 * (Q_{Eshunt})] / Q_{CO}$$

(6) Arterial oxygen venous content (C_aO_2)

The C_aO_2 was calculated based on the pulmonary shunt as following:

$$C_aO_2 = C_cO_2 - Q_S/Q_T * (C_cO_2 – C_VO_2)$$

Q_S/Q_T already includes the physiological shunt, mainly constituted by the bronchial circulation. The C_cO_2 is calculated based on the oxygen alveolar partial pressure:

$$P_AO_2 = FiO_2 * (P_{atm} – P_{H2O}) – PaCO_2 / RQ$$

Where P_{atm} is the barometric pressure (~ 690mmHg), RQ is the respiratory quotient (~ 0.8), and P_{H_2O} was considered as 47mmHg.

Considering the partial capillary pressure of oxygen equal to the P_AO_2, and hemoglobin oxygen saturation of 100%, C_CO_2 was defined as:

$$C_CO_2 = 0.0031 * P_AO_2 + 1.36 * Hb * 1$$

Combining those three last equations, the C_aO_2 was calculated as following:

$$C_aO_2 = [0.0031 * (FiO_2 * 643 – PaCO_2 / 0.8) + 1.36 * Hb * 1] - Q_S/Q_T * \{[0.0031 * (FiO_2 * 643 – PaCO_2 / 0.8) + 1.36 * Hb * 1] – [(C_{Post}O_2 * Q_{ECMOeff}) + C_VO_2 * Q_{Eshunt}] / Q_{CO} \}$$

(7) Arterial oxygen saturation ($SatO_2$)

$SatO_2$ was calculated as following:

$$SatO_2 = C_aO_2 / (1.36 * Hb)$$

After this point, the mathematical circuit reentries, closing the loop.

1.2.3. Looping and iteractions to reach the equilibrium

For starting equilibrium cycle of mathematical circuit (Figure 7S), the arterial saturation at the time of ECMO initiation (64%) was used. For any new variable tested, the circuit was recycled until reaching the equilibrium. In order to assure the equilibrium of the $SatO_2$, twenty iteractions (cycles) were tested (see the appendix to check the script).

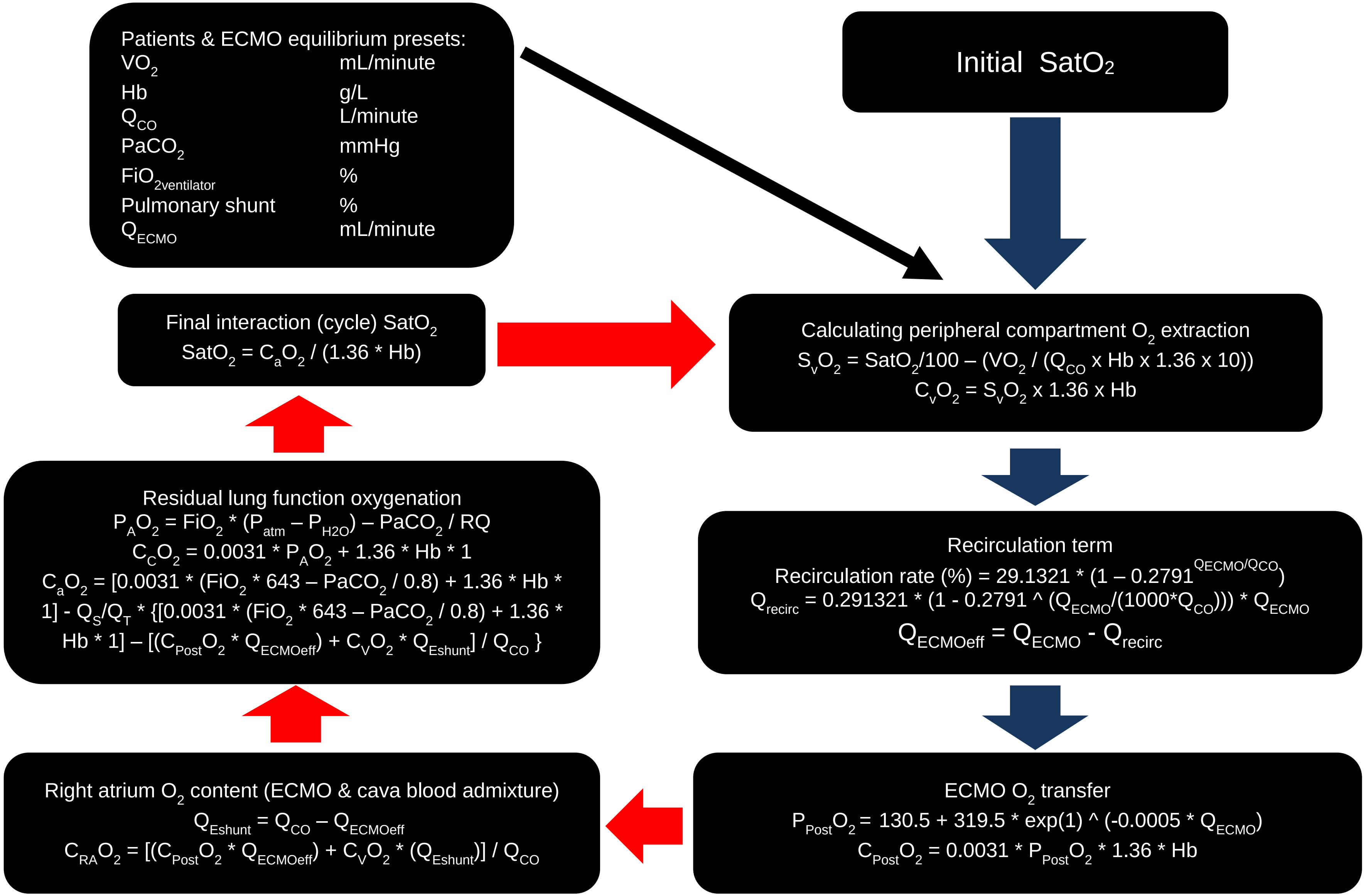
Patients & ECMO equilibrium presets:
VO_2 mL/minute
Hb g/L
Q_{CO} L/minute
$PaCO_2$ mmHg
$FiO_{2ventilator}$ %
Pulmonary shunt %
Q_{ECMO} mL/minute
Initial $SatO_2$
Final interaction (cycle) $SatO_2$
$SatO_2 = C_aO_2 / (1.36 * Hb)$
Calculating peripheral compartment O_2 extraction
$S_vO_2 = SatO_2/100 – (VO_2 / (Q_{CO} x Hb x 1.36 x 10))$
$C_vO_2 = S_vO_2 x 1.36 x Hb$
Residual lung function oxygenation
$P_AO_2 = FiO_2 * (P_{atm} – P_{H2O}) – PaCO_2 / RQ$
$C_cO_2 = 0.0031 * P_AO_2 + 1.36 * Hb * 1$
$C_aO_2 = [0.0031 * (FiO_2 * 643 – PaCO_2 / 0.8) + 1.36 * Hb * 1] - Q_S/Q_T * \{[0.0031 * (FiO_2 * 643 – PaCO_2 / 0.8) + 1.36 * Hb * 1] – [(C_{Post}O_2 * Q_{ECMOeff}) + C_VO_2 * Q_{Eshunt}] / Q_{CO} \}$
Recirculation term
Recirculation rate (%) = $29.1321 * (1 – 0.2791^{QECMO/QCO})$
$Q_{recirc} = 0.291321 * (1 - 0.2791 ^ (Q_{ECMO}/(1000*Q_{CO}))) * Q_{ECMO}$
$Q_{ECMOeff} = Q_{ECMO} - Q_{recirc}$
Right atrium O_2 content (ECMO & cava blood admixture)
$Q_{Eshunt} = Q_{CO} – Q_{ECMOeff}$
$C_{RA}O_2 = [(C_{Post}O_2 * Q_{ECMOeff}) + C_VO_2 * (Q_{Eshunt})] / Q_{CO}$
ECMO O_2 transfer
$P_{Post}O_2 = 130.5 + 319.5 * exp(1) ^ (-0.0005 * Q_{ECMO})$
$C_{Post}O_2 = 0.0031 * P_{Post}O_2 * 1.36 * Hb$

Figure 7S - Visual inspection of each step described above closing the iteractions. Twenty cycles (iteractions) were considered to reach the equilibrium. ECMO - extracorporeal membrane oxygenation; VO_2 – oxygen consumption; Hb – hemoglobin; Q_{CO} - cardiac output; $PaCO_2$ - partial pressure of carbon dioxide; $FiO_{2ventilator}$ - Ventilator fraction of inspired oxygen; Q_{ECMO} - extracorporeal membrane oxygenation blood flow; $SatO_2$ - oxygen saturation; C_aO_2- arterial content of oxygen; P_AO_2 - alveolar oxygen partial pressure; FiO_2 - fraction of inspired oxygen; P_{atm} - atmospheric pressure; P_{H2O} - partial pressure of water; RQ - respiratory coefficient; C_cO_2 - pulmonary alveolar capillary blood oxygen content; Q_S/Q_T - shunt fraction; $C_{post}O_2$ - post-extracorporeal membrane oxygenation oxygen blood content; $Q_{ECMOeff}$ - ECMO effective blood flow; C_vO_2 - venous oxygen blood content; Q_{Eshunt} - extracorporeal membrane oxygenation parallel cava blood flow (cava blood flow without extracorporeal membrane oxygenation); Q_{CO} - cardiac output; $C_{RA}O_2$ - right atrium oxygen content; $C_{post}O_2$ - post-extracorporeal membrane oxygenation oxygen blood content; S_vO_2 - Oxygen mixed venous saturation; Q_{recirc} - extracorporeal membrane oxygenation recirculation blood flow; $P_{Post}O_2$ - Oxygen partial pressure post-ECMO.

Some basic simulations were done to test the model consistency and stability. A clinical scenario is shown below, in which a patient is initiating ECMO support. The clinical variables are depicted in the graphs' legend. Three different hemoglobin levels were simulated to clarify the freedom of the model. The oxygen saturation and transfer are shown:

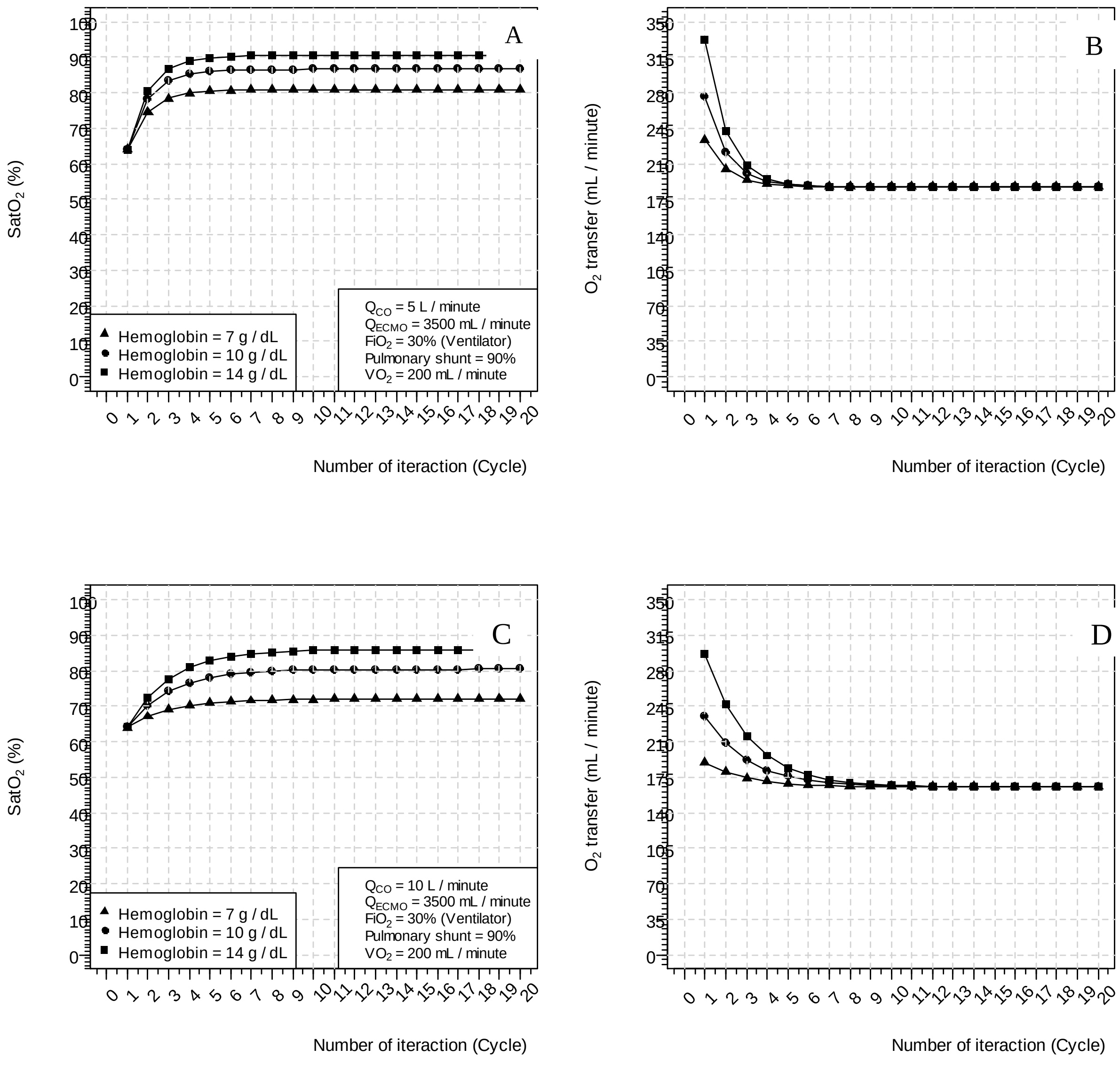

Figure 8S - A patient soon after extracorporeal membrane oxygenation initiation, already ultraprotectivelly ventilated is shown in **Panels A and B**, this patient presented an elevation of cardiac output to 10L/minute and the new clinical scenario with hypoxemia is shown in **Panels C and D**. $SatO_2$ - oxygen saturation; Q_{CO} - cardiac output; Q_{ECMO} - extracorporeal membrane oxygenation blood flow; FiO_2 - fraction of inspired oxygen; VO_2 - oxygen consumption.

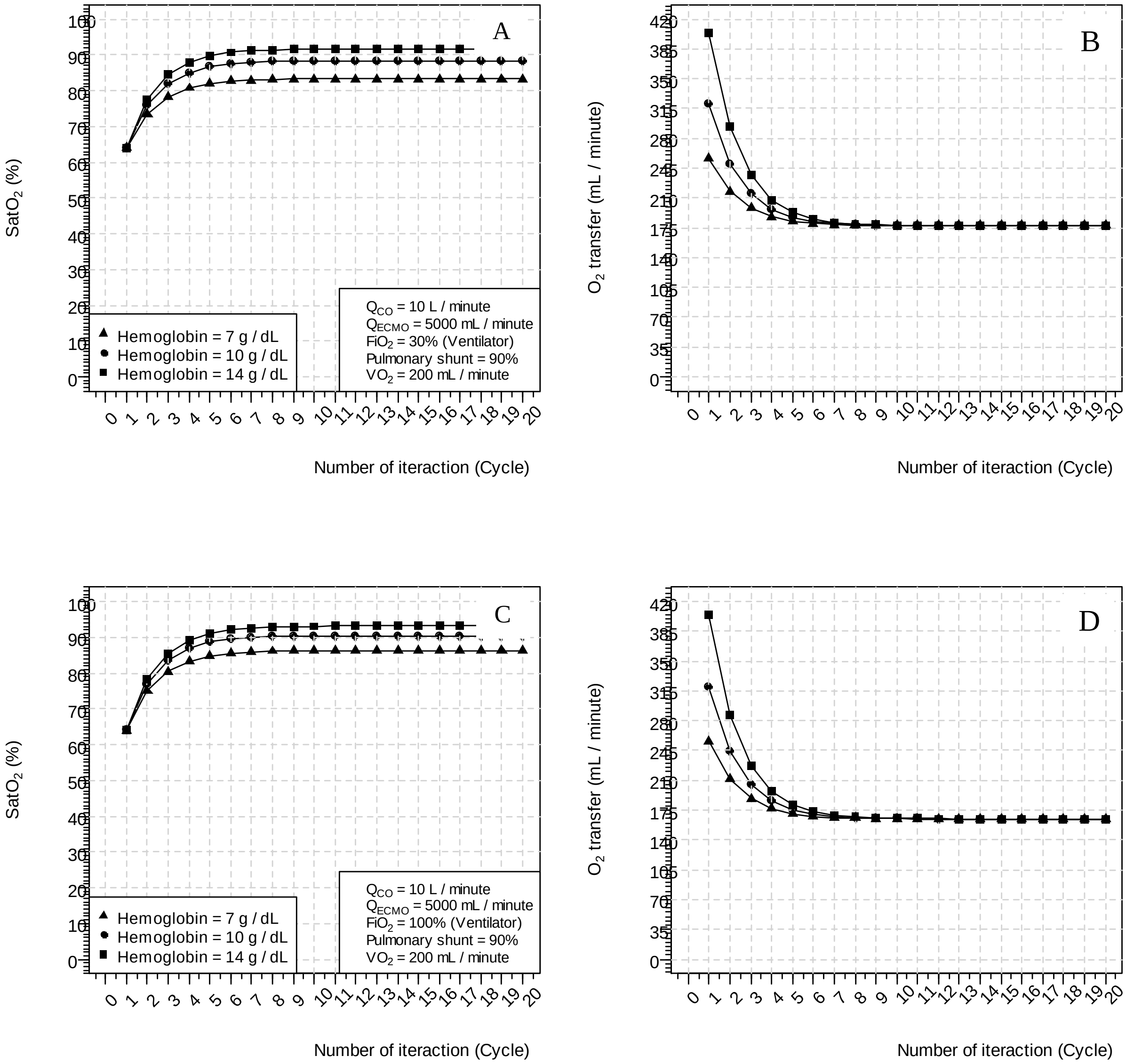

Figure 9S - As the patient was hypoxemic, the extracorporeal membrane oxygenation blood flow was increased to 5000 mL/minute as shown in **Panels A and B**, the ventilator fraction of inspired oxygen was then increased to 100% as shown in **Panels C and D**. $SatO_2$ – oxygen saturation; Q_{CO} - cardiac output; Q_{ECMO} - extracorporeal membrane oxygenation blood flow; FiO_2 - fraction of inspired oxygen; VO_2 - oxygen consumption.

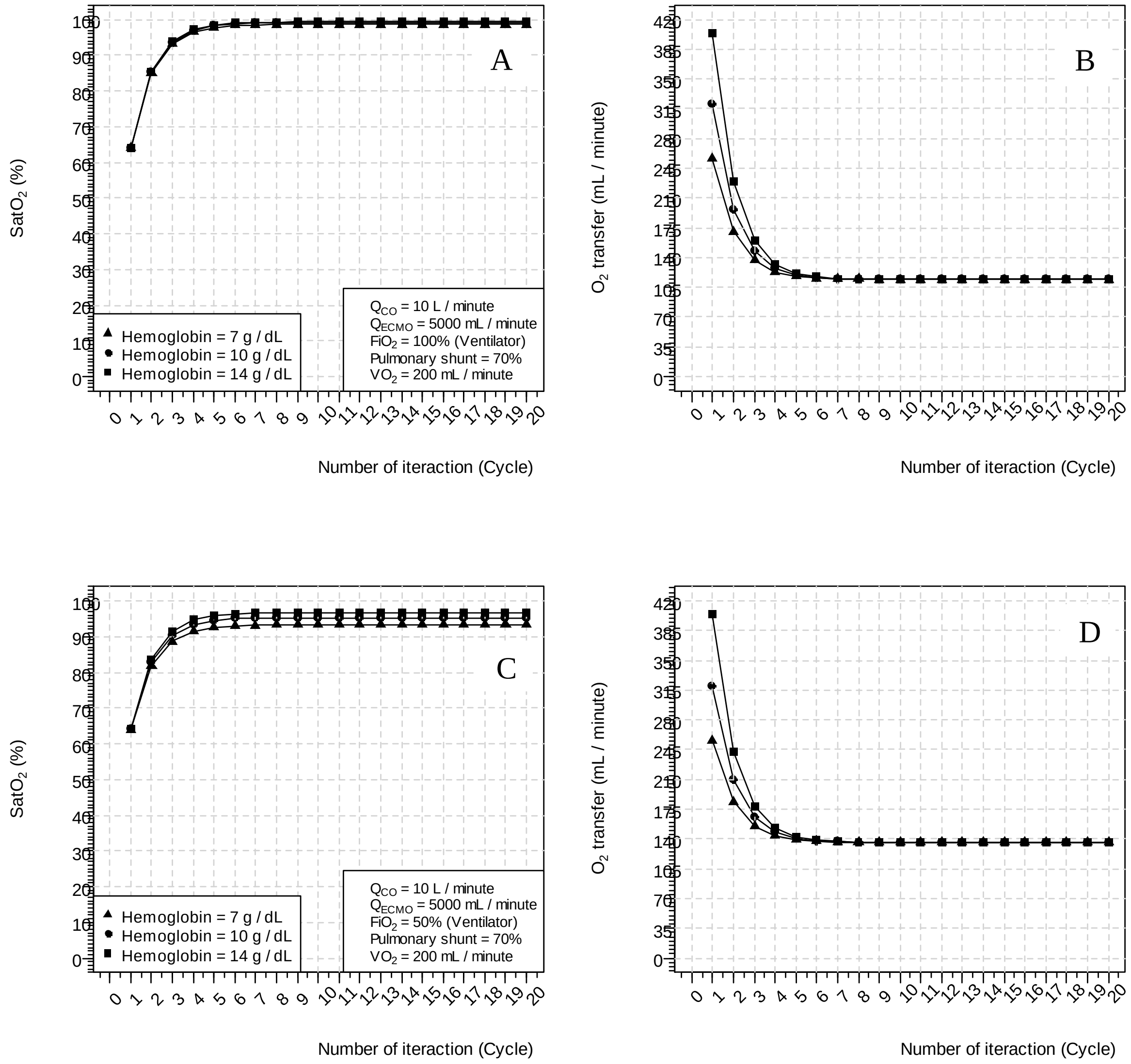

Figure 10S - The lungs of the patient were recruited with a high positive end-expiratory pressure, and the pulmonary shunt decreased to 70% as shown in **Panels A and B**, the ventilator fraction of inspired oxygen was then decreased to 70% as shown in **Panels C and D**. $SatO_2$ - oxygen saturation; Q_{CO} - cardiac output; Q_{ECMO} - extracorporeal membrane oxygenation blood flow; FiO_2 - fraction of inspired oxygen; VO_2 - oxygen consumption.

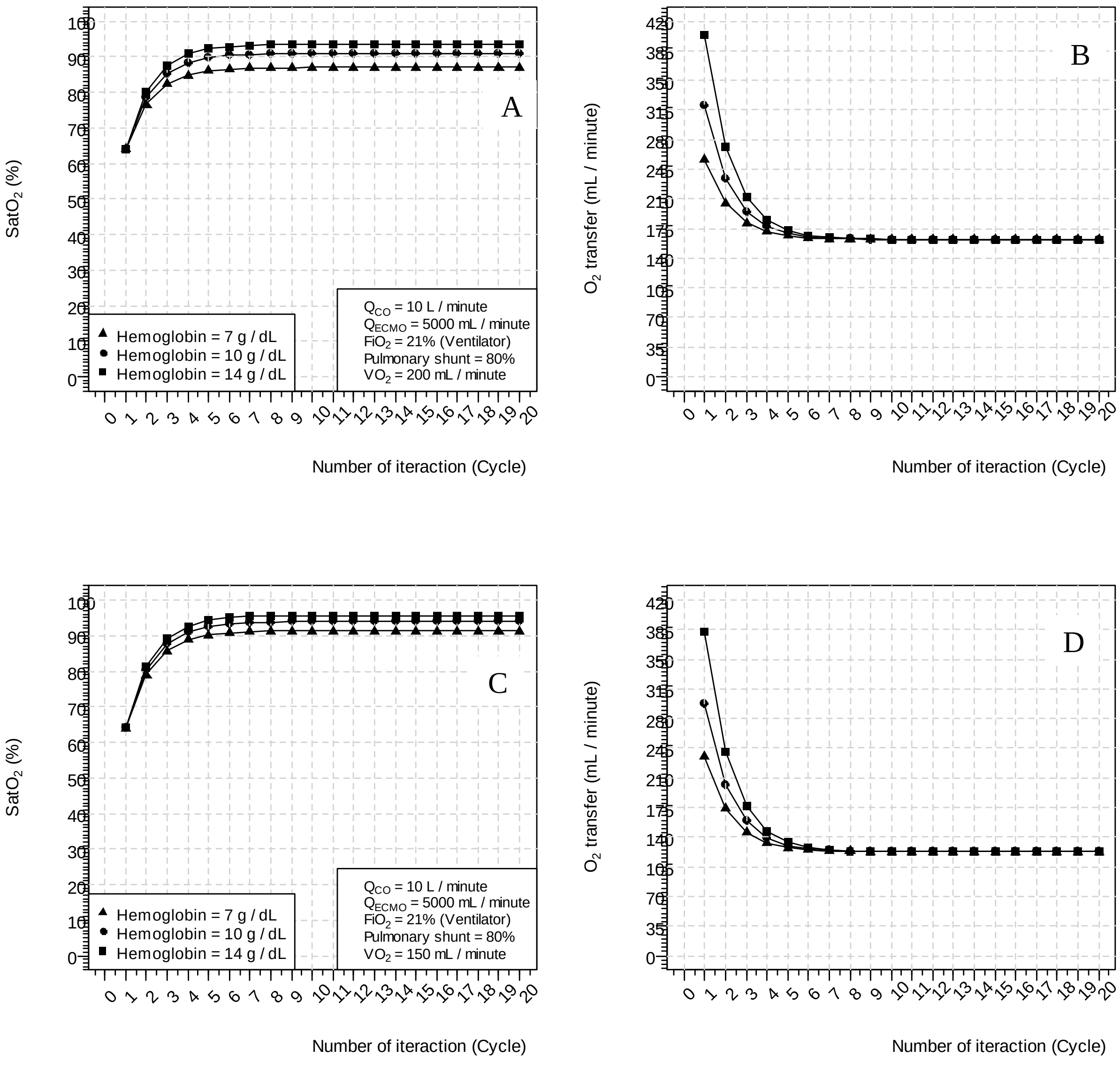

Figure 11S - With the oxygenation improvement, the fraction of inspired oxygen was further decreased to 21%, however the lungs de-recruited and pulmonary shunt increased to 80% as shown in **Panels A and B**, the patient clinical condition improved and the VO_2 decreased to 150mL/minute as shown in **Panels C and D**. $SatO_2$ - oxygen saturation; Q_{CO} - cardiac output; Q_{ECMO} - extracorporeal membrane oxygenation blood flow; FiO_2 - fraction of inspired oxygen; VO_2 - oxygen consumption.

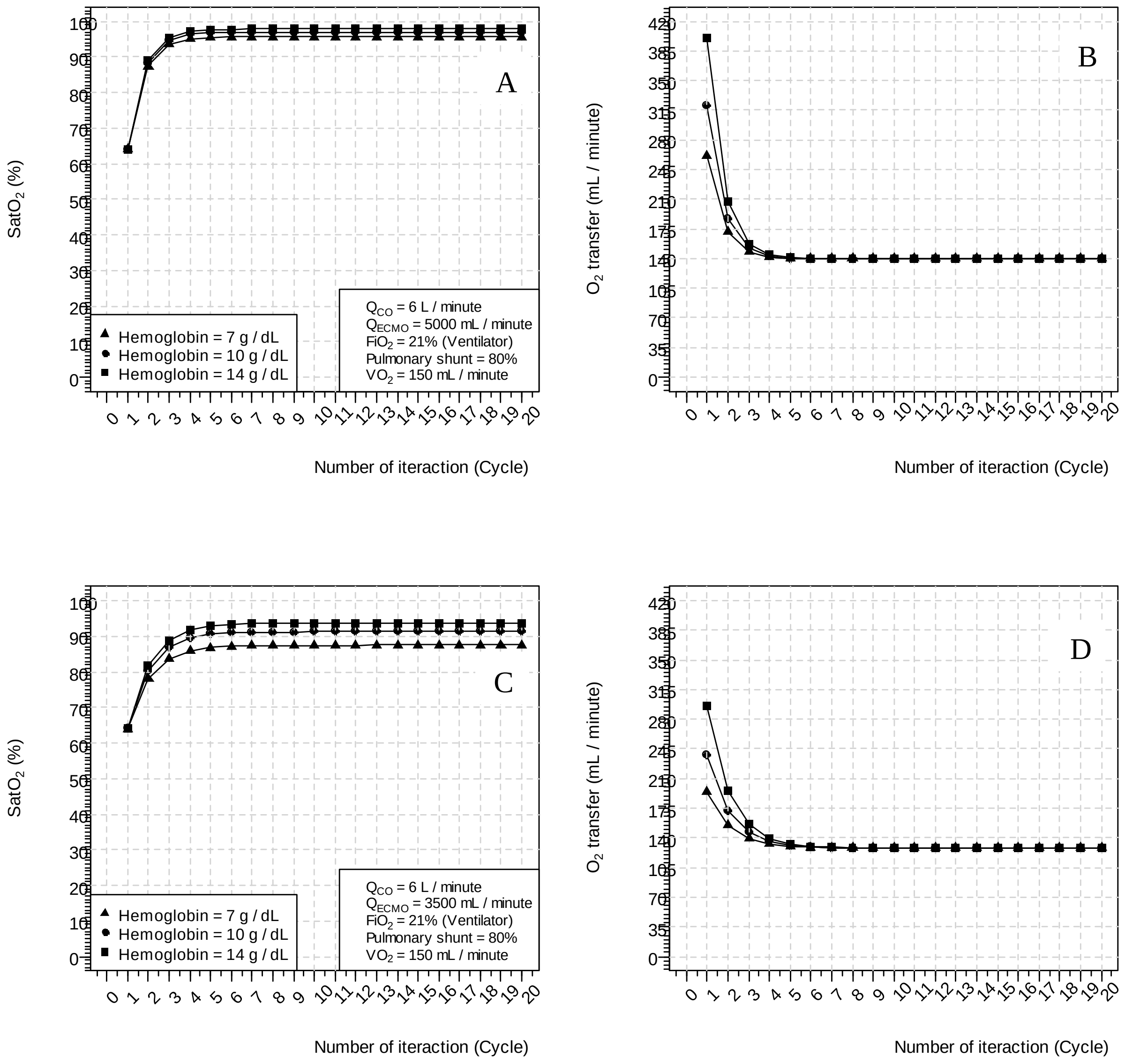

Figure 12S - Now with patient's improvement the cardiac output decreased to 6 L/minute as shown in **Panels A and B**, due to the patient oxygenation improvement, the extracorporeal membrane oxygenation blood flow was reduced to 3500mL/minute as shown in **Panels C and D**. $SatO_2$ - oxygen saturation; Q_{CO} - cardiac output; Q_{ECMO} - extracorporeal membrane oxygenation blood flow; FiO_2 - fraction of inspired oxygen; VO_2 - oxygen consumption.

The behavior of equilibrated values was compatible with ECMO-supported patients at the bedside.

1.3. Arterial carbon dioxide and total amount of carbon dioxide transfer

The CO_2 modeling was also based on femoral – jugular venous-venous configuration. The patient – ECMO coupling was considered as in the figure 2S.

1.3.1. Standard formulas used

Additionally to the 1.2.1 item, the blood CO_2 content was calculated using the Douglas et al. formula, in which the CO_2 content in three compartments – dissolved in plasma, as bicarbonate and linked to hemoglobin –, were considered.[17]

- pK = 6.086 + (0.042 * (7.4 - pH)) + ((38 - temperature) * 0.00472 + (0.00139 * (7.4 - pH)))
- s (CO_2 solubility) = 0.0307 + (0.00057 * (37 - temp)) + (0.00002 * $(37 - temp)^2$)
- Plasma CO_2 = 2.226 * s * P_LCO_2 * (1 + $10^{(ph-pk)}$)
- Blood local oxygen content (C_LCO_2) = (1 - ((0.0289 * Hb) / (3.352 - 0.456 * ($SatO_2$ / 100) * (8.142 - pH)))) * Plasma CO_2

1.3.2. Creating mathematical terms of the carbon dioxide modeling

(1) The recirculation term was used as described in the 1.2.2 item.

As described in the methods section, the patient had a P_aCO_2 = 62mmHg just before ECMO initiation.

(2) Initial $PvCO_2$ was modeled using a predetermined VCO_2, which will add CO_2 (CCO_{2added}) to the arterial blood resulting in the C_vCO_2 (mL of CO_2 / 100mL of blood).

The VCO_2 will be carried out by blood in a minute by a volume of blood equivalent to Q_{CO}. Therefore the CCO_{2added} was calculated as following:

$$CCO_{2added} = VCO_2 / (Q_{CO} * 10)$$

Taking into account the C_aCO_2, calculated as already described in the 1.3.1 item, the C_vCO_2 was calculated as following:

$$C_vCO_2 = C_aCO_2 + CCO_{2added}$$

Still using the Douglas formula rearranged,[17] the P_vCO_2 was calculated as following:

$$P_vCO_2 = \text{Plasma } CO_2 / (2.226 * s * (1 + 10^{(ph-pk)}))$$

The venous pH was considered as 0.02 lower than the arterial pH, as described in septic patients.[18]

Testing the consistency of P_vCO_2, the following graphs were built.

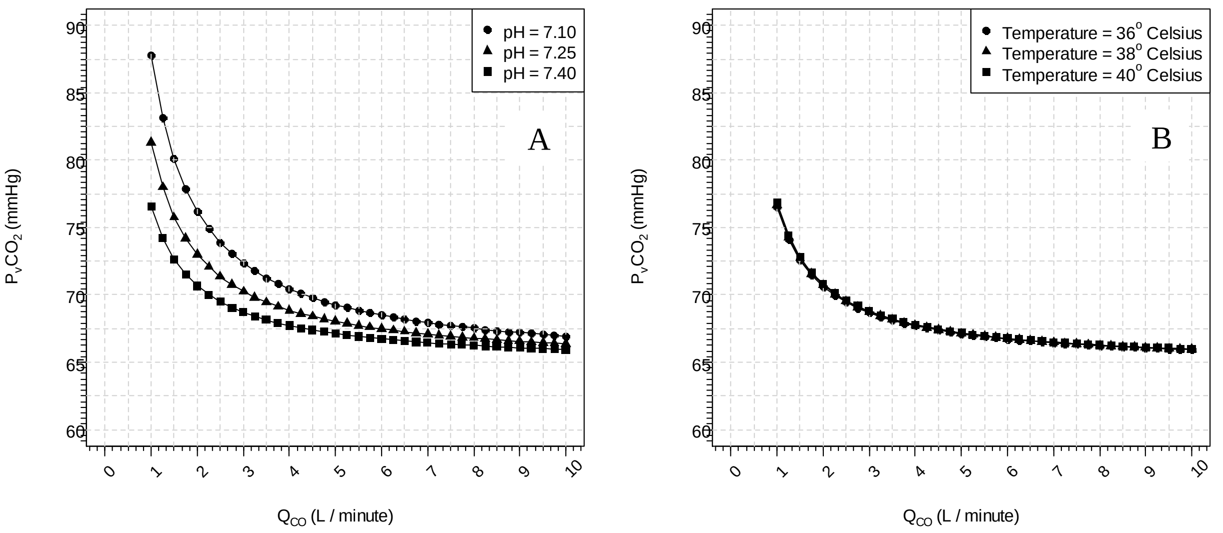

Figure 13S - The P_vCO_2 behavior with progressive cardiac output elevation with fixed values of VCO_2 = 150mL/minute, hemoglobin = 10g/dL, and initial partial pressure of carbon dioxide of 62mmHg. **Panels A** shows this behavior with different pHs (fixed temperature = 37° Celsius) and **Panel B** shows this behavior with different body temperatures (fixed pH = 7.40). P_vCO_2 - mixed venous carbon dioxide partial pressure. VCO_2 – carbon dioxide production

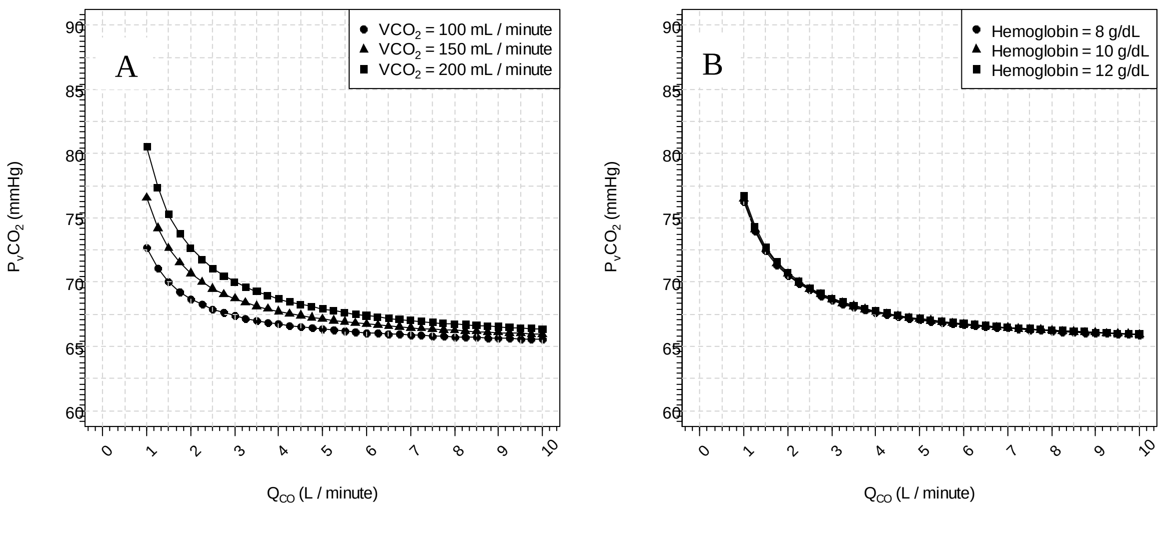

Figure 14S -The P_vCO_2 behavior with progressive cardiac output elevation with fixed values of pH = 7.40, temperature = 37° Celsius, and initial partial pressure of carbon dioxide of 62mmHg. **Panel A** shows this behavior with different VCO_2s (fixed hemoglobin = 10g/dL) and **Panel B** shows this behavior with different hemoglobins (fixed VCO_2 = 150mL/minute). VCO_2 – carbon dioxide production.

(3) ECMO CO_2 transfer (CO_{2t})

The CO_{2t} was calculated as described previously by our group.[15] The CO_{2t} depends on sweep gas flow, hemoglobin level, Q_{ECMO}, and P_vCO_2. The CO_{2t} formula used was:

$$CO_{2t} = (Q_{ECMO} * 0.017) + (sweep * 32.91) + (P_vCO_2 * 1.192) - (Hb * 3.478)$$

(4) CO_2 ECMO removal (CO_{2r}) and $C_{post}CO_2$

Using the same rationale of volume / time used to calculate P_vCO_2, the CO_{2r} was calculated as:

$$CO_{2r} = (Q_{ECMO} * C_vCO_2 / 100) - CO_{2t}$$

The $C_{post}CO_2$ was then calculated as:

$$C_{post}CO_2 = CO_{2r} * 100 / Q_{ECMO}$$

(5) Modeling $C_{RA}CO_2$

The $C_{RA}CO_2$ was calculated through the proportional C_vCO_2 and $C_{post}CO_2$ admixture. The proportion of each CO_2 content was extracted from Q_{ECMO} and Q_{Eshunt}.

$$C_{RA}CO_2 = (Q_{ECMO} / (Q_{CO} * 1000) * C_{post}CO_2) + (Q_{Eshunt} / (Q_{CO} * 1000) * C_vCO_2)$$

(6) Lung ventilation and decarboxylation

The effective lung minute ventilation and decarboxylation was modeled based on end-tidal CO_2 ($EtCO_2$). Barometric pressure was considered 690mmHg. The proportion of CO_2 removed in the residual function of native lungs was considered as $EtCO_2$ / 690. The volume of CO_2 removed by the native lungs (CO_{2rl}) was then considered as:

$$CO_{2rl} = \text{Respiratory rate} * \text{tidal volume} * EtCO_2 / 690$$

The final C_cCO_2 was calculated based on the proportional flow through the ventilated lung regions as:

$$C_cCO_2 = \{[C_{RA}CO_2 * (Q_{CO} - Q_{Lshunt})] - CO_{2rl}\} / [100 * (Q_{CO} - Q_{Lshunt})]$$

(7) Modeling P_aCO_2

The C_aCO_2 was calculated based on the proportion of Q_{Lshunt} as:

$$C_aCO_2 = (C_{RA}CO_2 * Q_{Lshunt} / Q_{CO}) + C_cCO_2 * (Q_{Lshunt} - Q_{CO}) / Q_{CO}$$

At last, P_aCO_2 was calculated using the Douglas method:

$$P_aCO_2 = \text{Plasma } CO_2 / (2.226 * s * (1 + 10^{(ph\text{-}pk)}))$$

This value of P_aCO_2 re-enters the loop until stabilization.

1.3.3. Looping and iteractions to reach the equilibrium

For starting the equilibrium cycle of the mathematical circuit (Figure 15S), the arterial saturation at the time of ECMO initiation of 64% and an initial P_aCO_2 of 62mmHg

were used. For any new variable tested, the circuit was recycled until reaching the equilibrium. In order to assure the equilibrium of the P_aCO_2, eight hundred (cycles) were tested (see the appendix to check the script).

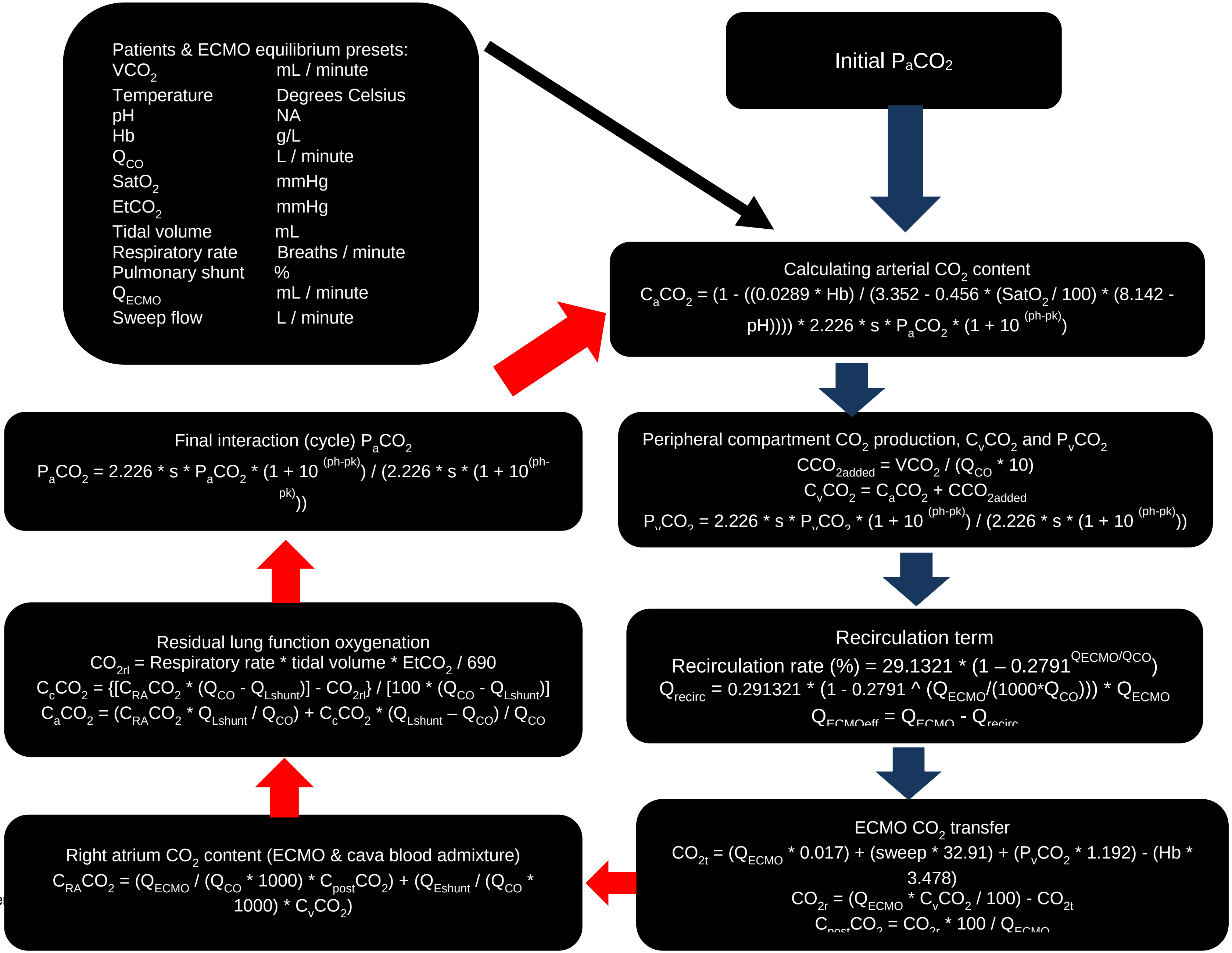
Patients & ECMO equilibrium presets:
VCO_2 mL / minute
Temperature Degrees Celsius
pH NA
Hb g/L
Q_{CO} L / minute
$SatO_2$ mmHg
$EtCO_2$ mmHg
Tidal volume mL
Respiratory rate Breaths / minute
Pulmonary shunt %
Q_{ECMO} mL / minute
Sweep flow L / minute
Initial P_aCO_2
Calculating arterial CO_2 content
C_aCO_2 = (1 - ((0.0289 * Hb) / (3.352 - 0.456 * ($SatO_2$ / 100) * (8.142 - pH)))) * 2.226 * s * P_aCO_2 * (1 + 10 $^{(ph-pk)}$)
Peripheral compartment CO_2 production, C_vCO_2 and P_vCO_2
CCO_{2added} = VCO_2 / (Q_{CO} * 10)
C_vCO_2 = C_aCO_2 + CCO_{2added}
P_vCO_2 = 2.226 * s * P_vCO_2 * (1 + 10 $^{(ph-pk)}$) / (2.226 * s * (1 + 10 $^{(ph-pk)}$))
Recirculation term
Recirculation rate (%) = 29.1321 * (1 – 0.2791$^{QECMO/QCO}$)
Q_{recirc} = 0.291321 * (1 - 0.2791 ^ (Q_{ECMO}/(1000*Q_{CO}))) * Q_{ECMO}
$Q_{ECMOeff}$ = Q_{ECMO} - Q_{recirc}
ECMO CO_2 transfer
CO_{2t} = (Q_{ECMO} * 0.017) + (sweep * 32.91) + (P_vCO_2 * 1.192) - (Hb * 3.478)
CO_{2r} = (Q_{ECMO} * C_vCO_2 / 100) - CO_{2t}
$C_{post}CO_2$ = CO_{2r} * 100 / Q_{ECMO}
Right atrium CO_2 content (ECMO & cava blood admixture)
$C_{RA}CO_2$ = (Q_{ECMO} / (Q_{CO} * 1000) * $C_{post}CO_2$) + (Q_{Eshunt} / (Q_{CO} * 1000) * C_vCO_2)
Residual lung function oxygenation
CO_{2rl} = Respiratory rate * tidal volume * $EtCO_2$ / 690
C_cCO_2 = {[$C_{RA}CO_2$ * (Q_{CO} - Q_{Lshunt})] - CO_{2rl}} / [100 * (Q_{CO} - Q_{Lshunt})]
C_aCO_2 = ($C_{RA}CO_2$ * Q_{Lshunt} / Q_{CO}) + C_cCO_2 * (Q_{Lshunt} – Q_{CO}) / Q_{CO}
Final interaction (cycle) P_aCO_2
P_aCO_2 = 2.226 * s * P_aCO_2 * (1 + 10 $^{(ph-pk)}$) / (2.226 * s * (1 + 10$^{(ph-pk)}$))

Figure 15S - Visual inspection of each step described above closing the iteractions. Eight hundred cycles (iteractions) were considered to reach the equilibrium. ECMO - extracorporeal membrane oxygenation; Hb - hemoglobin; Q_{CO} - cardiac output; $SatO_2$ – oxygen saturation; $EtCO_2$ - end-tidal carbon dioxide partial pressure; Q_{ECMO} - extracorporeal membrane oxygenation blood flow; $PaCO_2$ - partial pressure of carbon dioxide; C_aCO_2 - arterial content of carbon dioxide; CO_2 - carbon dioxide; C_vCO_2 - venous carbon dioxide blood content; PvCO2 – mixed venous carbon dioxide partial pressure; CCO_{2added} – added carbon dioxide content; VCO_2 - carbon dioxide production; C_vCO_2 - venous carbon dioxide blood content; Q_{ECMO} - extracorporeal membrane oxygenation blood flow; Q_{recirc} - extracorporeal membrane oxygenation recirculation blood flow; $Q_{ECMOoff}$ - ECMO efficient blood flow; CO_{2t} - carbon dioxide transfer; CO_{2r} - carbon dioxide removed by native lungs; $C_{post}CO_2$ - post-extracorporeal membrane oxygenation carbon dioxide blood content; $C_{RA}O_2$ - right atrium oxygen content; C_cCO_2 - pulmonary alveolar capillary carbon dioxide blood content; $C_{RA}CO_2$ - right atrium carbon dioxide content; Q_{Lshunt} - pulmonary blood flow in shunt regions.

In order to test the model consistency and stability, as in the oxygenation model, some basic simulations were done. A clinical scenario is shown below, in which a patient is initiating ECMO support. The clinical variables are depicted in the graphs' legend. Three different hemoglobin levels were simulated to clarify the freedom of the model. The $PaCO_2$ and transfer are shown:

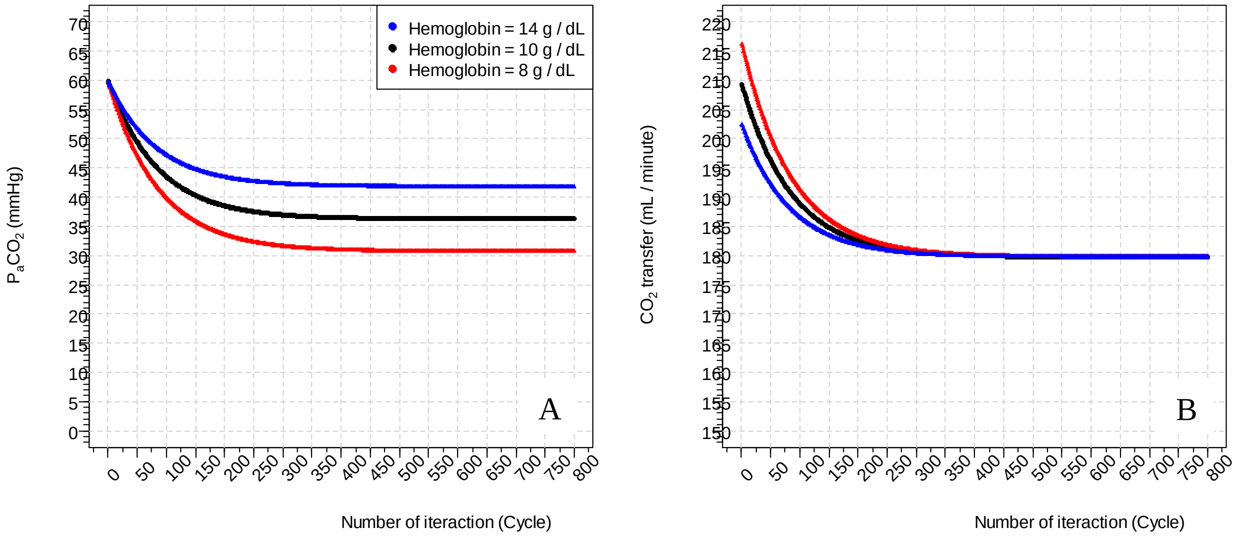

Figure 16S - Partial pressure of carbon dioxide (**Panel A**) after 800 iteractions of the mathematical model with three different values of hemoglobin and the respective carbon dioxide transfer (**Panel B**). P_aCO_2 - partial pressure of carbon dioxide.

Other controlled variables were:

Q_{CO} = 8 L/minute

VCO_2 = 180mL/minute

pH = 7.40

Temperature = 37ºC

Q_{ECMO} = 3500 mL/minute

Sweep gas flow = 3.5L/minute

Q_{Pshunt} = 95%

$EtCO_2$ = 2mmHg

Vt = 10mL

RR = 10 BPM

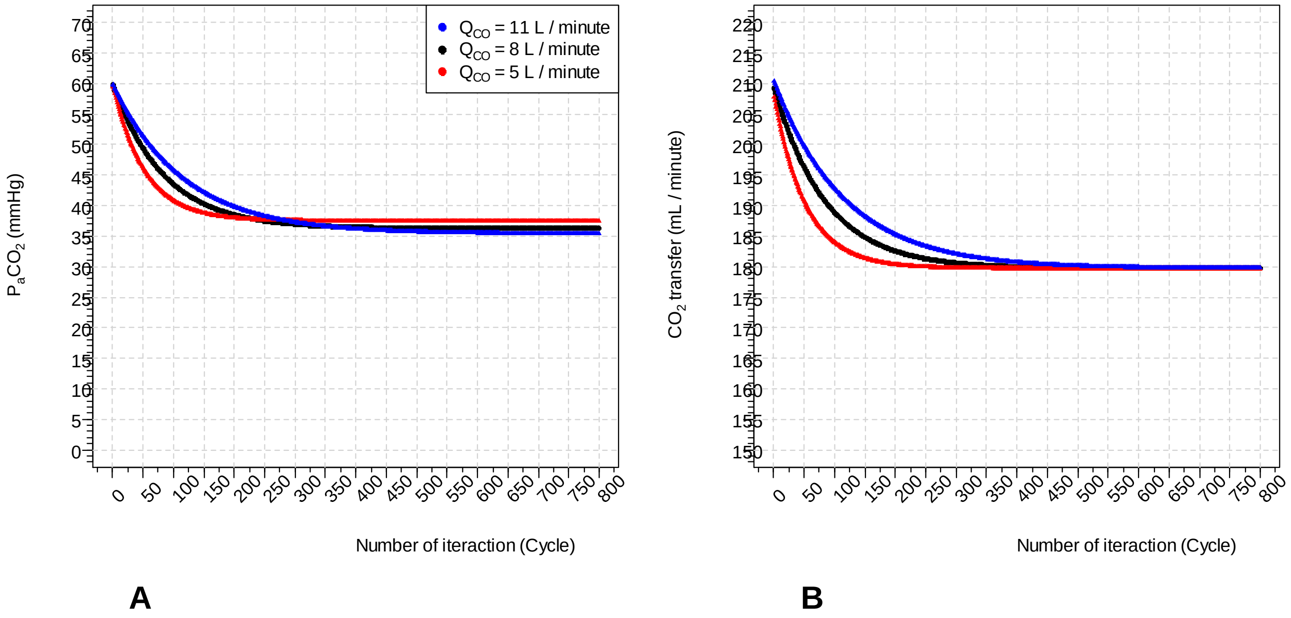

Figure 17S - Partial pressure of carbon dioxide (**Panel A**) after 800 iteractions of the mathematical model with three different values of cardiac output and the respective carbon dioxide transfer (**Panel B**). P_aCO_2 - partial pressure of carbon dioxide; Q_{CO} - cardiac output.

Other controlled variables were:

Hb = 10g/dL

VCO_2 = 180mL/minute

pH = 7.40

Temperature = 37° C

Q_{ECMO} = 3500mL/minute

Sweep gas flow = 3.5L/minute

Q_{Pshunt} = 95%

$EtCO_2$ = 2mmHg

Vt = 10mL

RR = 10 BPM

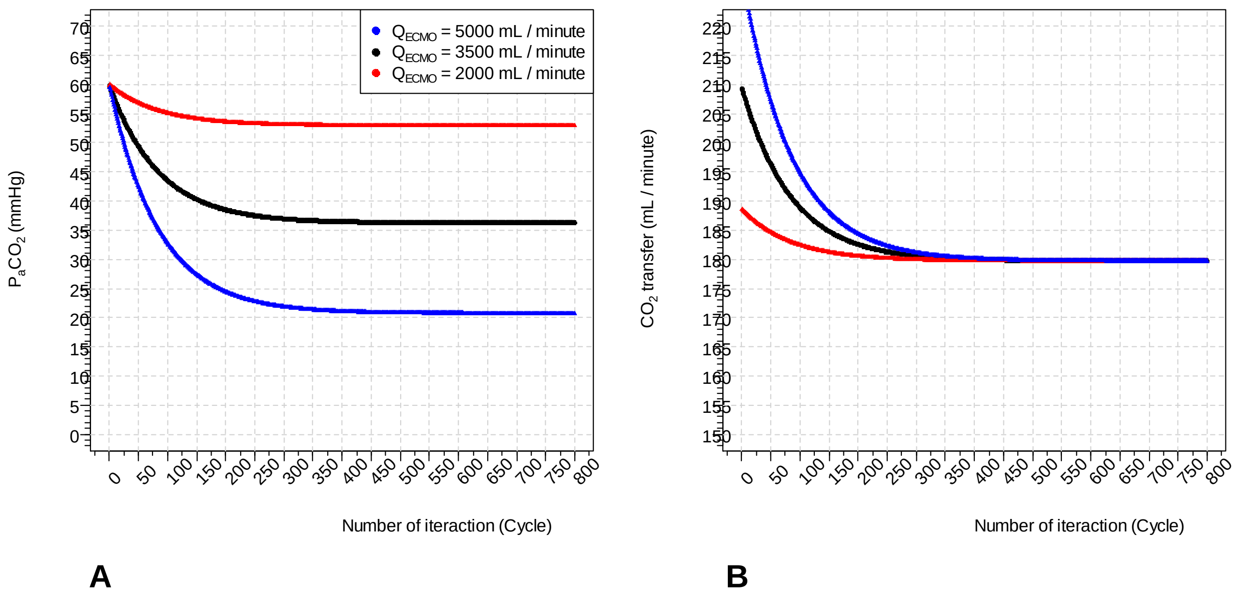

Figure 18S - Partial pressure of carbon dioxide (**Panel A**) after 800 iteractions of the mathematical model with three different values of extracorporeal membrane oxygenation blood flow and the respective carbon dioxide transfer (**Panel B**). P_aCO_2 - partial pressure of carbon dioxide; Q_{ECMO} - extracorporeal membrane oxygenation blood flow.

Other controlled variables were:

Hb = 10g/dL

VCO_2 = 180mL/minute

pH = 7.40

Temperature = 37ºC

Q_{CO} = 8 L/minute

Sweep gas flow = 3.5L/minute

Q_{Pshunt} = 95%

$EtCO_2$ = 2mmHg

Vt = 10mL

RR = 10 BPM

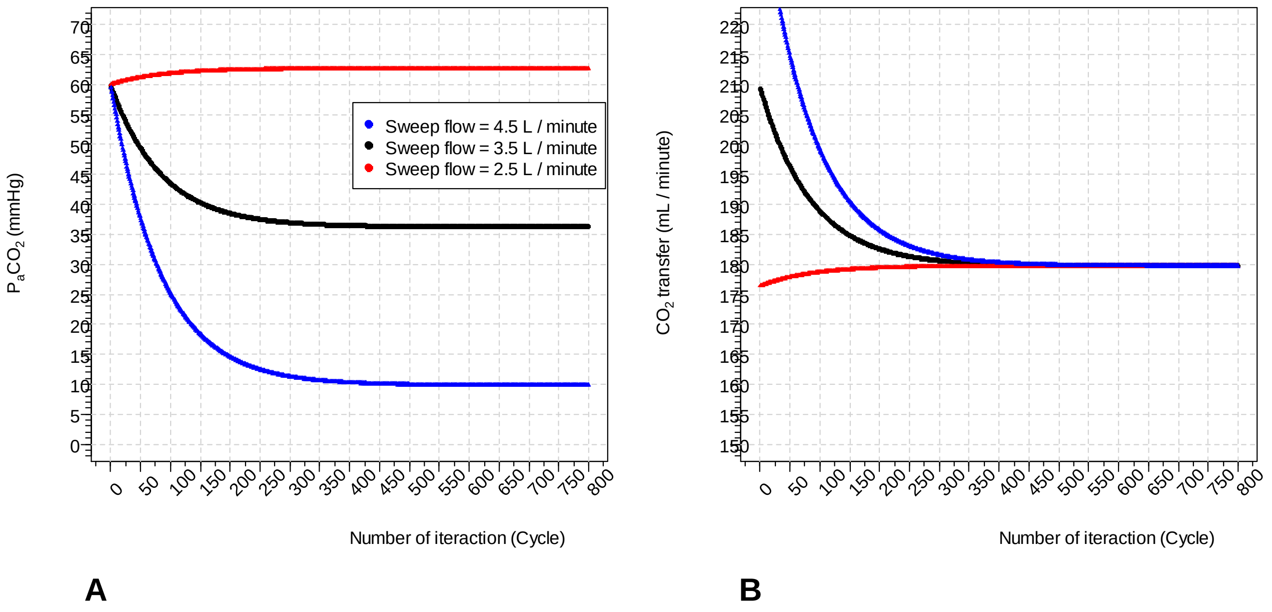

Figure 19S - Partial pressure of carbon dioxide (**Panel A**) after 800 iteractions of the mathematical model with three different values of sweep flow and the respective carbon dioxide transfer (**Panel B**). P_aCO_2 - partial pressure of carbon dioxide.

Other controlled variables were:

Hb = 10g/dL

VCO_2 = 180mL/minute

pH = 7.40

Temperature = 37° C

Q_{CO} = 8L/minute

Q_{ECMO} = 3500mL/minute

Q_{Pshunt} = 95%

$EtCO_2$ = 2mmHg

Vt = 10mL

RR = 10 BPM

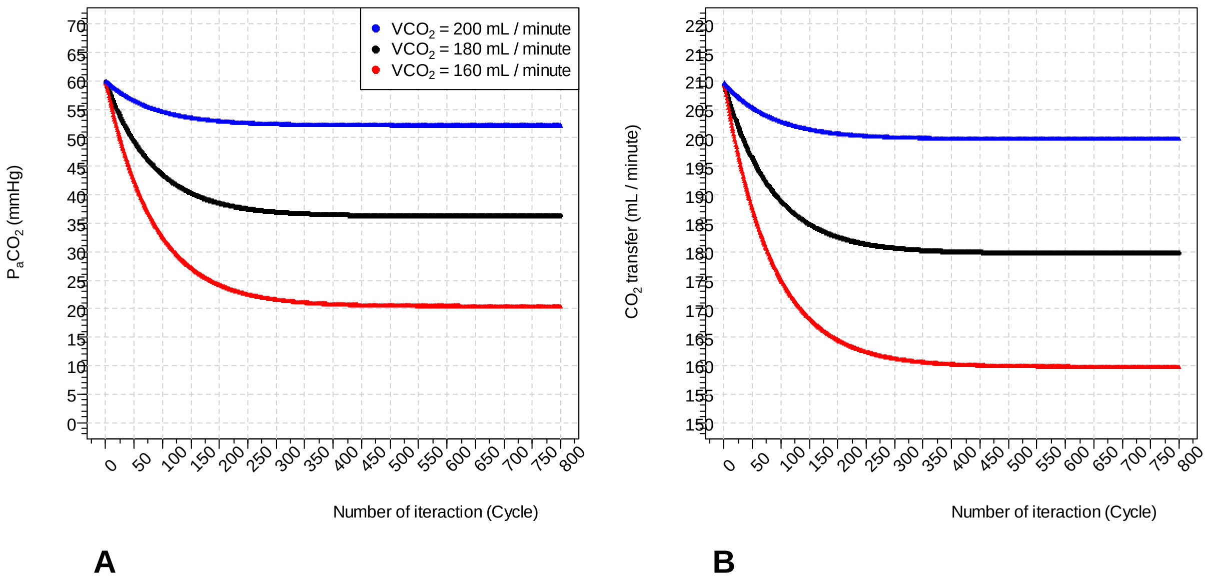

Figure 20S - Partial pressure of carbon dioxide (**Panel A**) after 800 iteractions of the mathematical model with three different values of VCO_2 and the respective carbon dioxide transfer (**Panel B**). P_aCO_2 - partial pressure of carbon dioxide; VCO_2 - carbon dioxide production.

Other controlled variables were:

Hb = 10g/dL

Sweep gas flow = 3.5L/minute

pH = 7.40

Temperature = 37ºC

Q_{CO} = 8L/minute

Q_{ECMO} = 3500mL/minute

Q_{Pshunt} = 95%

$EtCO_2$ = 2mmHg

Vt = 10mL

RR = 10 BPM

1.4. Oxygen partial pressure responsible for pulmonary vasoconstriction inhibition ($P_{stimulus}O_2$).

The pulmonary pressure during ARDS results from many factors, such as hypoxemia, hypercapnia, pH, cardiac output, and lung parenchyma collapse. The oxygen partial pressure responsible for modulation of pulmonary arterial pressure results from the alveolar (P_AO_2) and venous oxygen partial pressure (P_vO_2) as following:[19]

$$P_{stimulus}O_2 = P_AO_2^{0.68} + P_vO_2^{0.32}$$

The Q_{Pshunt} was taken into account considering the P_AO_2 in shunt regions as zero due to alveolar collapse or absence of fresh air. In this way, the final $P_{stimulus}O_2$ was calculated as:

$$P_{stimulus}O_2 = [(Q_{Pshunt} / Q_{CO}) * P_vO_2^{0.32}] + [(1 – (Q_{Pshunt} / Q_{CO})) * (P_AO_2^{0.68} + P_vO_2^{0.32})]$$

2. RESULTS

This section shows complementary analyses using the mathematical models.

2.1. Energy transfer from the ventilator to the lungs

The marginal effect of PEEP, driving pressure, RR, and I:E ratio on the mechanical power in ultraprotective ventilation are shown below. Other variables besides the variable of interest were kept as described in the clinical scenario in the methods section of the main manuscript.

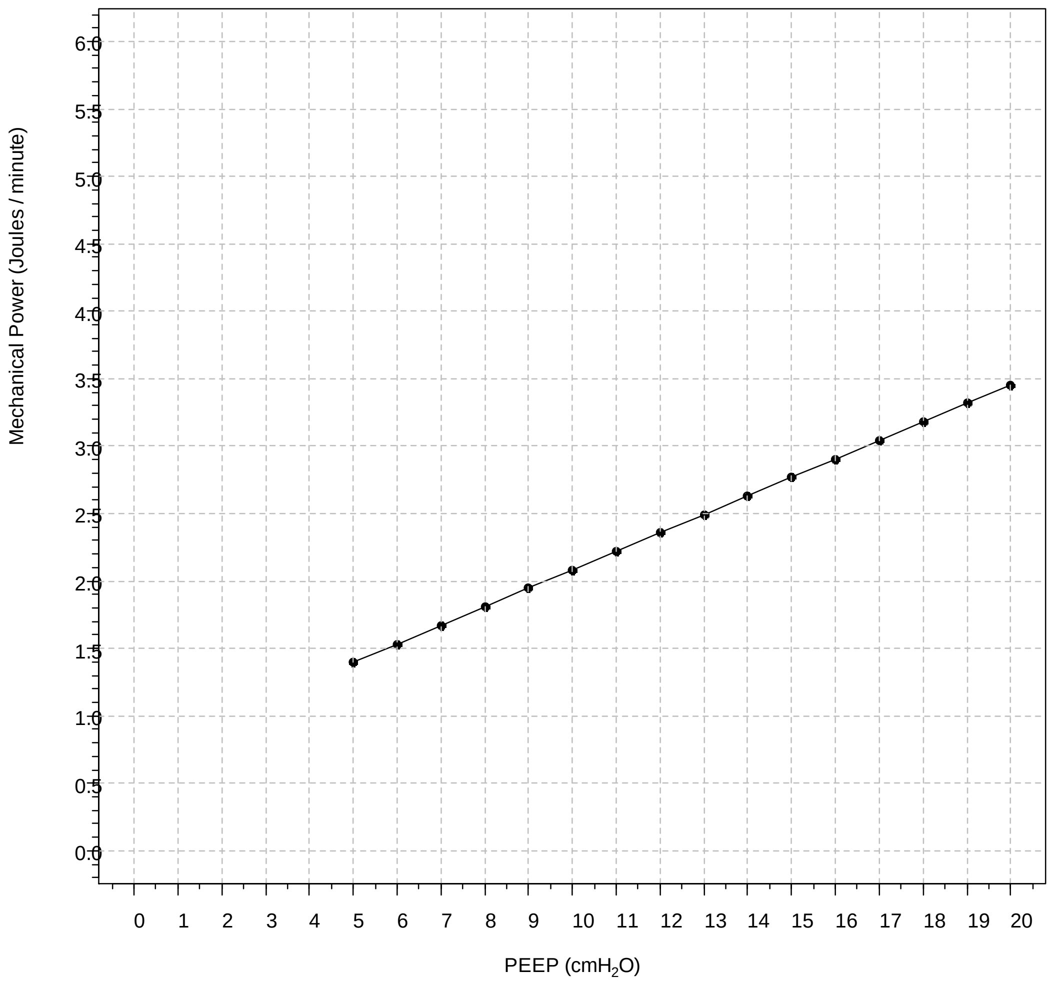

Figure 21S - Marginal effect of positive end-expiratory pressure on the mechanical power. The controlled variables were respiratory rate = 10 BPM, I:E = 1:2, driving pressure = 10cmH_2O, and static respiratory compliance = 14mL/cmH_2O. PEEP - positive end-expiratory pressure.

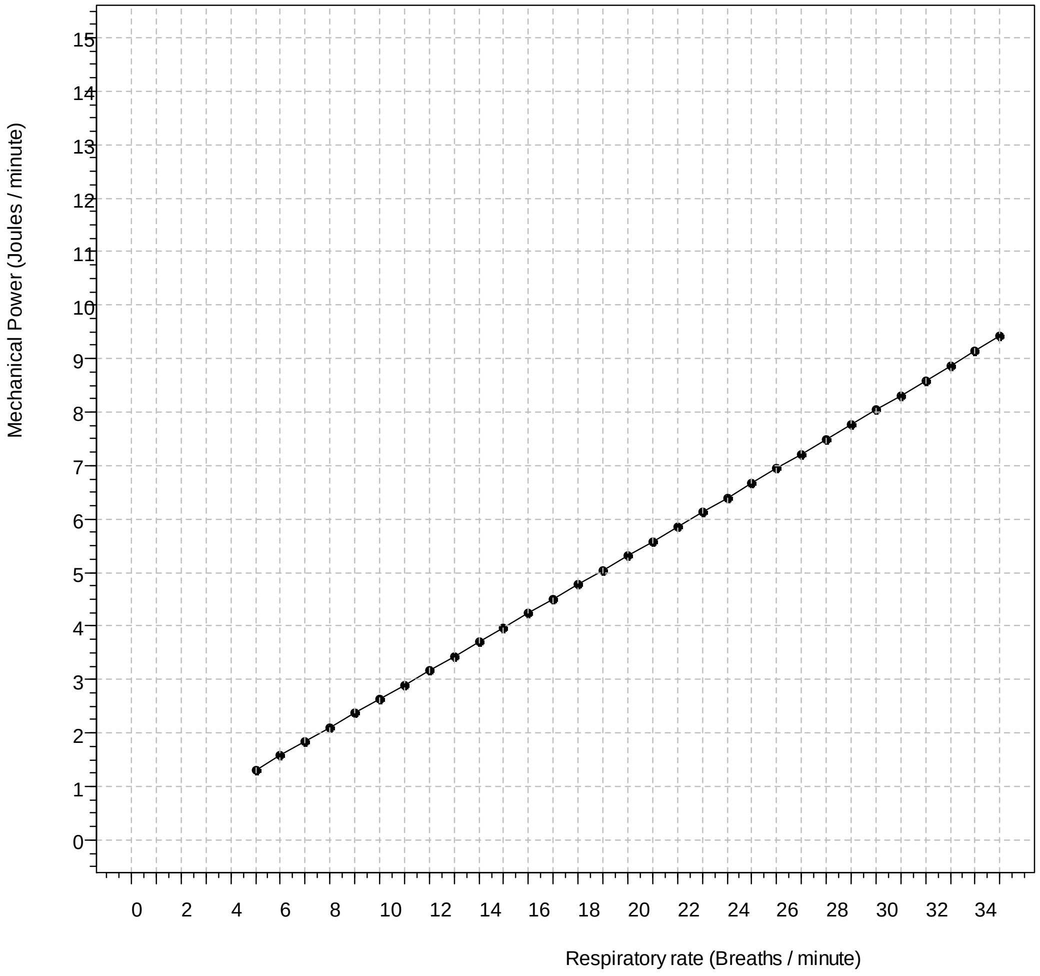

Figure 22S - Marginal effect of respiratory rate on the mechanical power. The controlled variables were positive end-expiratory pressure = 10cmH_2O, I:E = 1:2, driving pressure = 10cmH_2O, and static respiratory compliance = 14mL/cmH_2O.

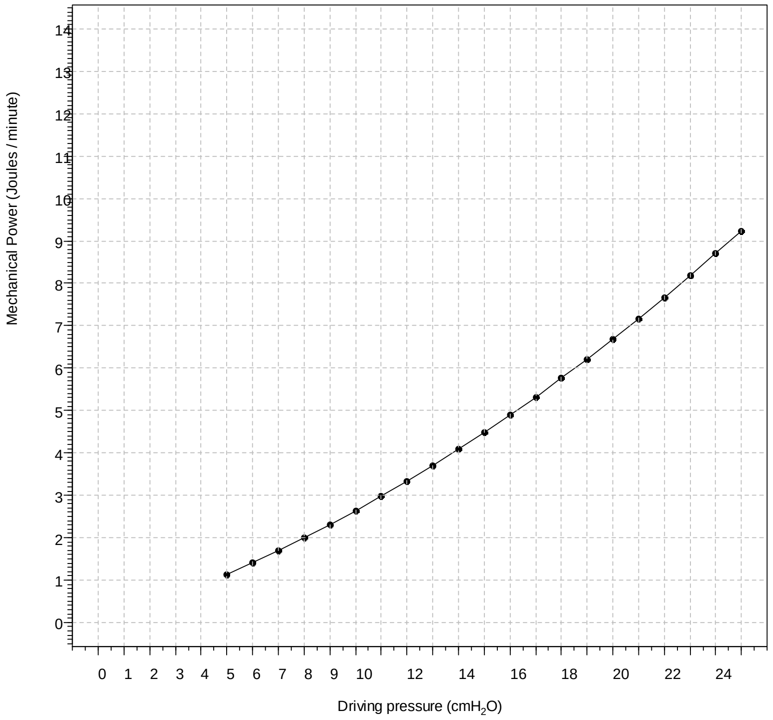

Figure 23S - Marginal effect of driving pressure on the mechanical power. The controlled variables were positive end-expiratory pressure = 10cmH_2O, I:E = 1:2, respiratory rate = 10 BPM, and static respiratory compliance = 14mL/cmH_2O.

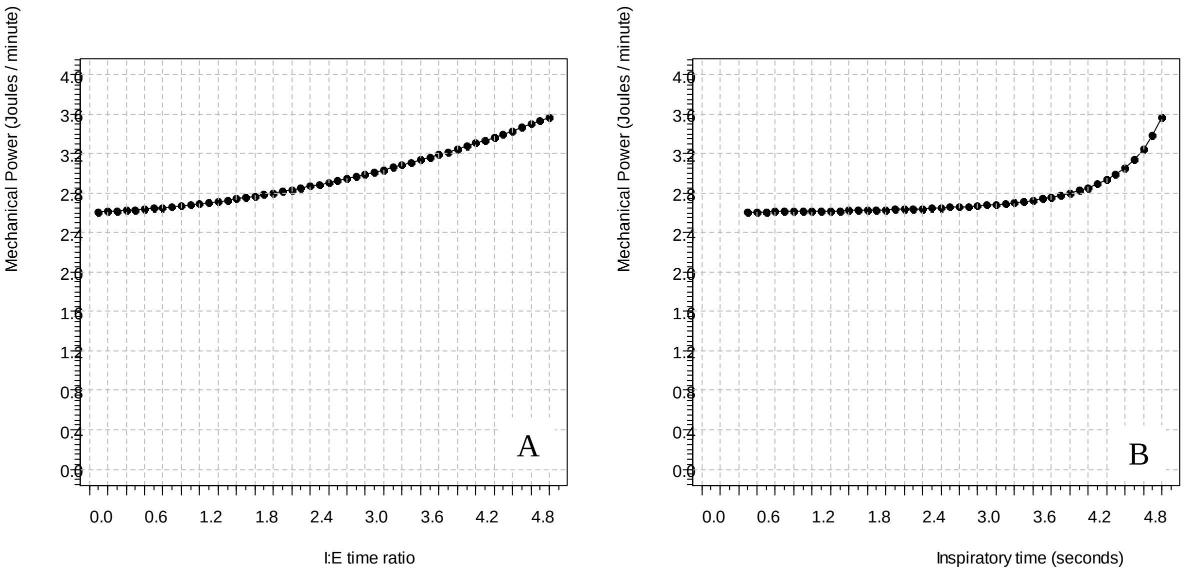

Figure 24S - Marginal effect of I:E ratio on the mechanical power. **Panel A** shows the association of I:E time ratio and mechanical power, and **Panel B** shows the association between inspiratory time in seconds with the mechanical power (with a fixed respiratory rate = 10 BPM). The controlled variables were positive end-expiratory pressure = 10cmH_2O, driving pressure = 10cmH_2O, respiratory rate = 10 BPM, and static respiratory compliance = 14mL/cmH_2O.

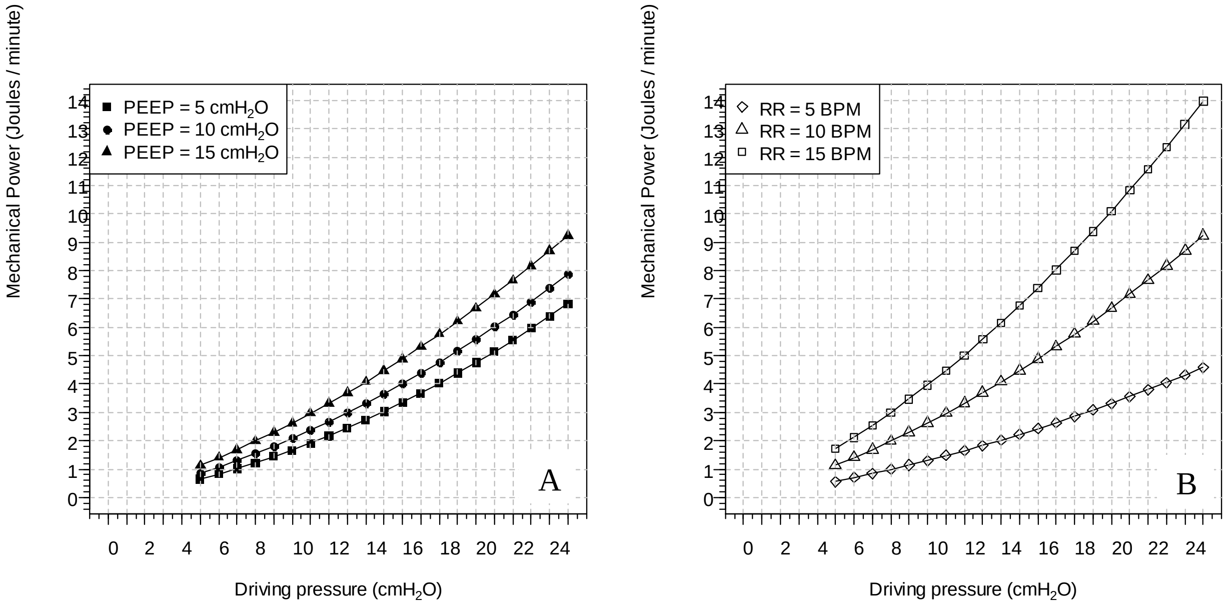

Figure 25S - Marginal effect of driving pressure on the mechanical power. **Panel A** shows the association between the plotted variables with different positive end-expiratory pressure levels (with a controlled respiratory rate = 10 BPM), and **Panel B** shows the association of plotted variables with different respiratory rates (with a controlled positive end-expiratory pressure = 10cmH_2O). Static respiratory compliance was 14mL/cmH_2O.

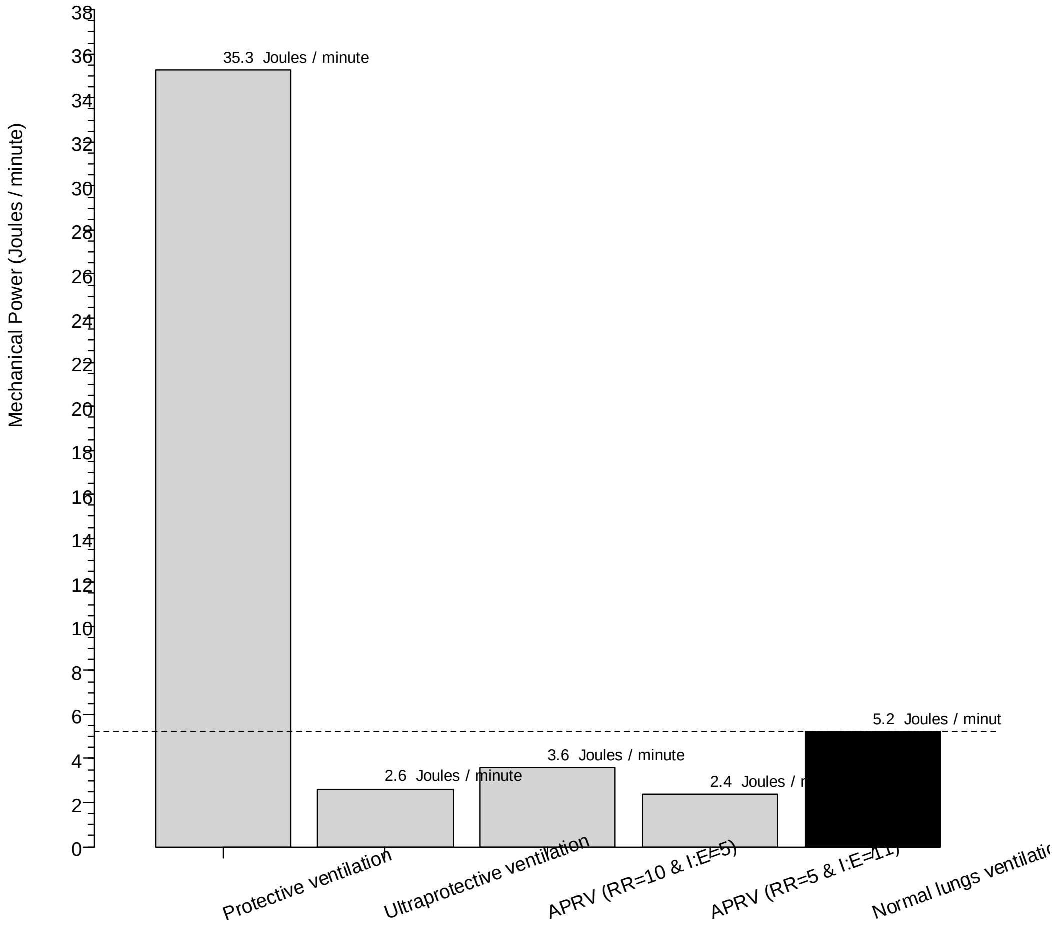

	Protective ventilation	Ultraprotective ventilation	APRV (RR = 10 & I:E = 5)	APRV (RR = 5 & I:E = 11)	Normal lung ventilation
Clinical scenario	ARDS	ARDS	ARDS	ARDS	General anesthesia
C_{st} (mL/cmH_2O)	18	14	14	14	100
R_{aw} (cmH_2O/L/second)	10	10	10	10	6
Tidal volume (mL)	360	14	14	14	480
Respiratory rate (BPM)	35	10	10	5	14
PEEP (cmH_2O)	18	14	14	14	5
I:E	1:2	1:2	5:1	11:1	1:2

Figure 26S - Mechanical power expressing the energy load per minute transferred from the mechanical ventilator to the lungs. APRV - airway pressure release ventilation; RR - respiratory rate; I:E – I/E time ratio.

Clinical scenario of the ARDS patient is shown in the methods text of the main manuscript. Normal lungs ventilation expresses the mechanical power of a healthy patient under general anesthesia for an elective surgery.

2.2. Arterial oxygenation and total amount of oxygen transfer

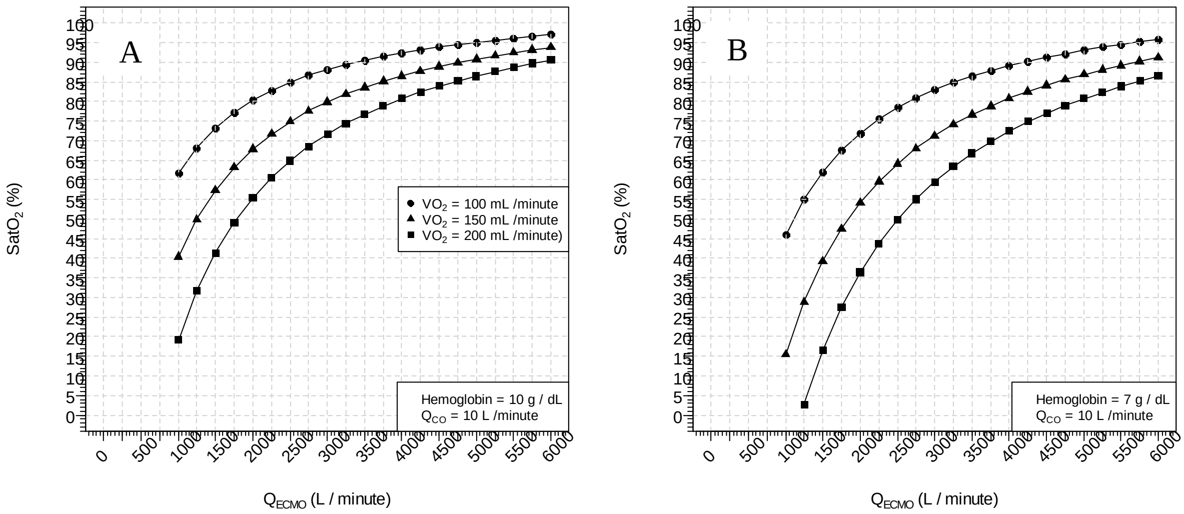

Figure 27S - Marginal effect of extracorporeal membrane oxygenation blood flow on oxygen saturation at different VO_2, with a fixed pulmonary blood flow in shunt regions of 95%. **Panel A** shows the effect with hemoglobin of 10g/dL and a cardiac output of 10L/minute, and **Panel B** shows the effect with hemoglobin of 7g/dL and cardiac output of 10L/minute. $SatO_2$ – oxygen saturation; VO_2 - oxygen consumption; Q_{CO} - cardiac output; Q_{ECMO} - extracorporeal membrane oxygenation blood flow.

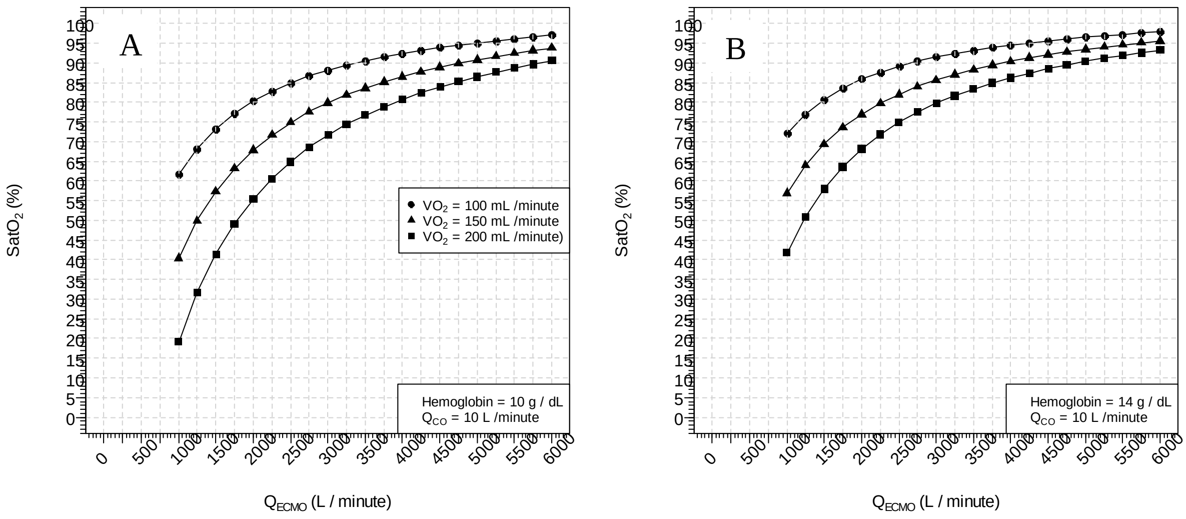

Figure 28S - Marginal effect of extracorporeal membrane oxygenation blood flow on oxygen saturation at different VO_2, with a fixed pulmonary blood flow in shunt regions of 95%. **Panel A** shows the effect with hemoglobin of 10g/dL and a cardiac output of 10L/minute, and **Panel B** shows the effect with hemoglobin of 14g/dL and cardiac output of 10L/minute. $SatO_2$ - oxygen saturation; VO_2 - oxygen consumption; Q_{CO} - cardiac output; Q_{ECMO} - extracorporeal membrane oxygenation blood flow.

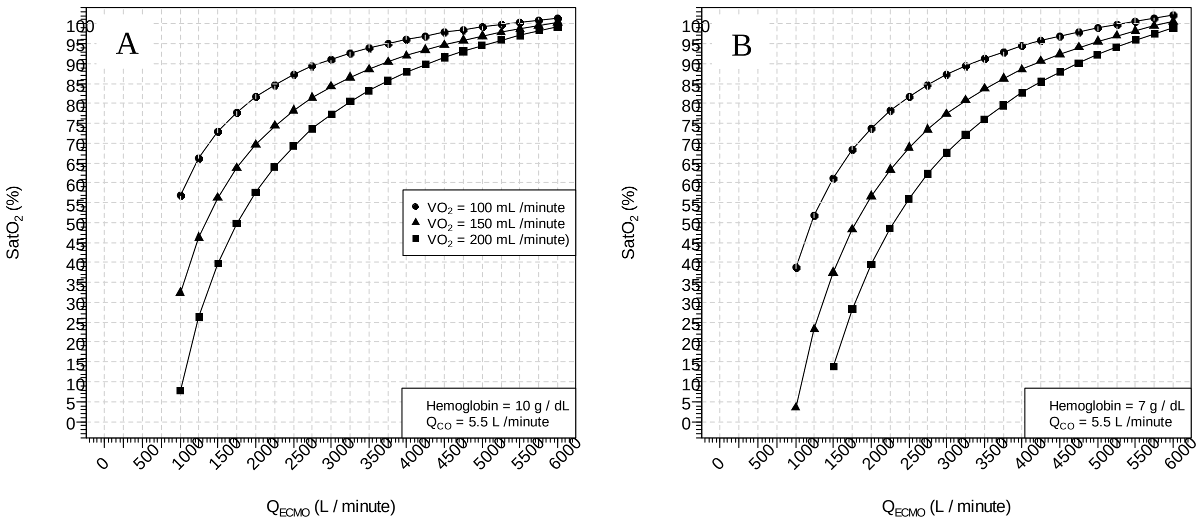

Figure 29S - Marginal effect of extracorporeal membrane oxygenation blood flow on oxygen saturation at different VO_2, with a fixed pulmonary blood flow in shunt regions of 95%. **Panel A** shows the effect with hemoglobin of 10g/dL and a cardiac output of 5.5L/minute, and **Panel B** shows the effect with hemoglobin of 7g/dL and cardiac output of 5.5L/minute. $SatO_2$ - oxygen saturation; VO_2 - oxygen consumption; Q_{CO} - cardiac output; Q_{ECMO} - extracorporeal membrane oxygenation blood flow.

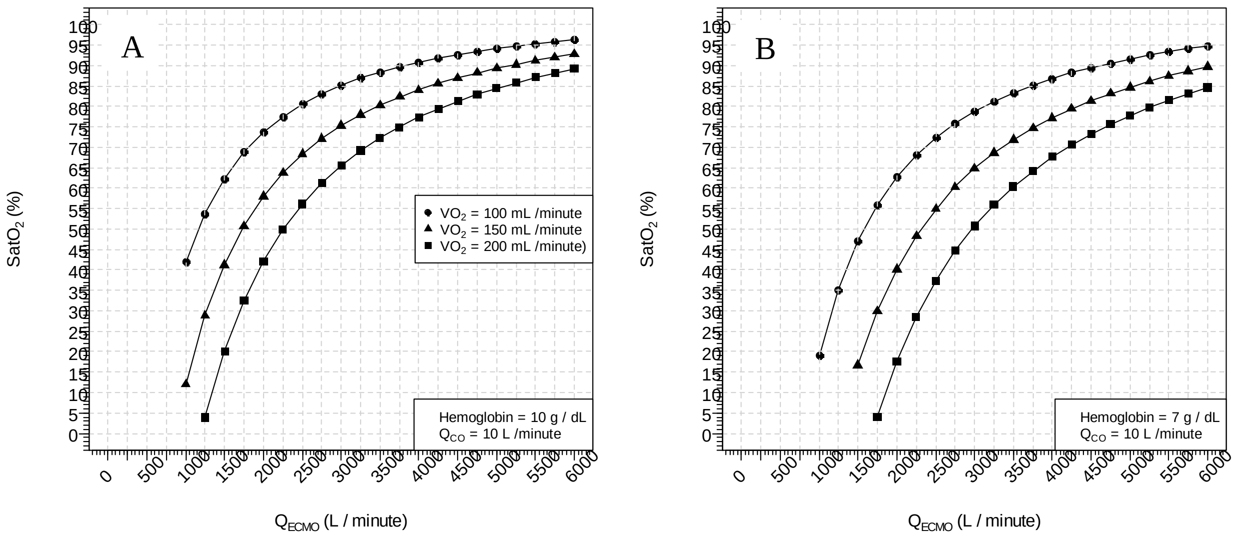

Figure 30S - Marginal effect of extracorporeal membrane oxygenation blood flow on oxygen saturation at different VO_2, with a fixed pulmonary blood flow in shunt regions of 100%. **Panel A** shows the effect with hemoglobin of 10g/dL and a cardiac output of 10L/minute, and **Panel B** shows the effect with hemoglobin of 7g/dL and cardiac output of 10L/minute. $SatO_2$ - oxygen saturation; VO_2 - oxygen consumption; Q_{CO} - cardiac output; Q_{ECMO} - extracorporeal membrane oxygenation blood flow.

2.3. Arterial carbon dioxide and total amount of carbon dioxide transfer

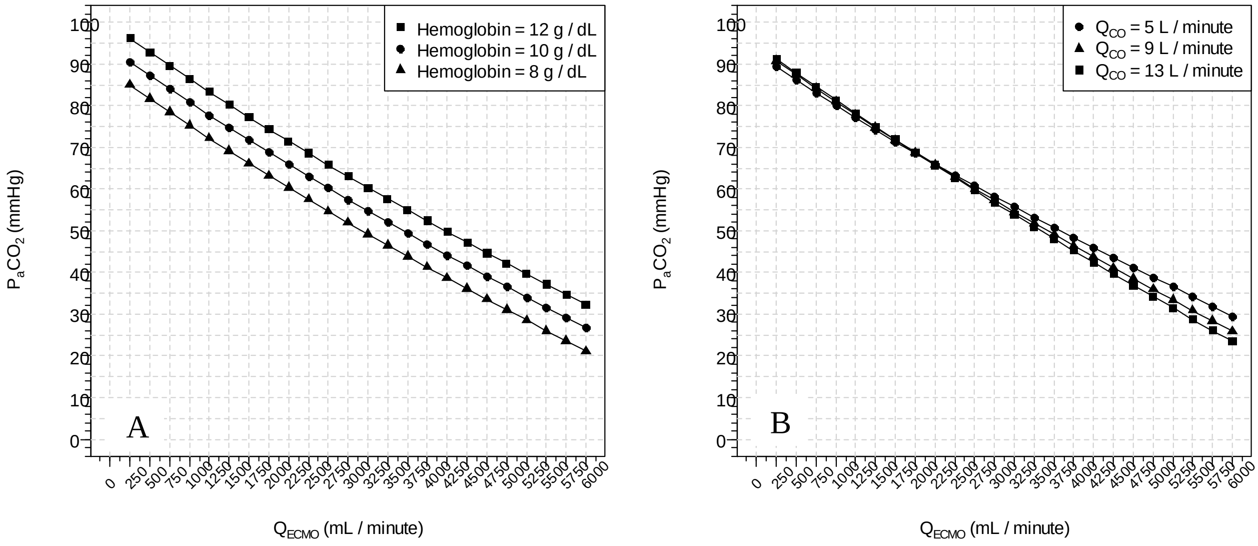

Figure 31S - Partial pressure of carbon dioxide behavior with progressive increments of extracorporeal membrane oxygenation blood flow with three different hemoglobin levels and a cardiac output = 8L/minute (**Panel A**), and three different cardiac output with fixed hemoglobin = 8g/dL (**Panel B**). $PaCO_2$ - partial pressure of carbon dioxide; Q_{ECMO} - extracorporeal membrane oxygenation blood flow; Q_{CO} - cardiac output.

Other controlled variables were:

VCO_2 = 200mL/minute

pH = 7.40

Temperature = 37ºC

Q_{CO} = 8L/minute

Q_{Pshunt} = 95%

$EtCO_2$ = 2mmHg

Vt = 10mL

RR = 10 BPM

2.4. Oxygen partial pressure responsible for pulmonary vasoconstriction inhibition ($P_{stimulus}O_2$)

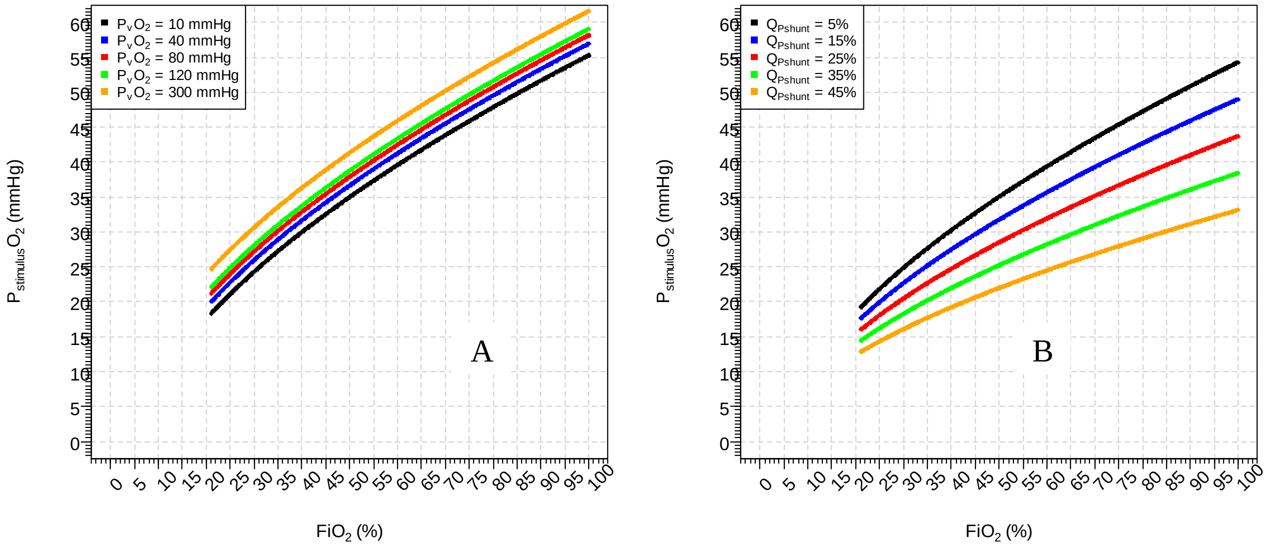

Figure 32S - $P_{stimulus}O_2$ behavior with progressive increments of fraction of inspired oxygen with different P_vO_2 levels (**Panel A**), and $P_{stimulus}O_2$ behavior with progressive increments of fraction of inspired oxygen with different Q_{Pshunt} levels (**Panel B**). $P_{stimulus}O_2$ - oxygen partial pressure responsible for hypoxic vasoconstriction; FiO_2 - fraction of inspired oxygen; P_vO_2 - mixed venous oxygen partial pressure; Q_{Pshunt} - shunt fraction.

3. REFERENCES

1. Chiumello D, Carlesso E, Cadringher P, Caironi P, Valenza F, Polli F, et al. Lung stress and strain during mechanical ventilation for acute respiratory distress syndrome. Am J Respir Crit Care Med. 2008;178(4):346-55.

2. Protti A, Votta E, Gattinoni L. Which is the most important strain in the pathogenesis of ventilator-induced lung injury: dynamic or static? Curr Opin Crit Care. 2014;20(1):33-8.

3. Protti A, Andreis DT, Monti M, Santini A, Sparacino CC, Langer T, et al. Lung stress and strain during mechanical ventilation: any difference between statics and dynamics? Crit Care Med. 2013;41(4):1046-55.

4. Gattinoni L, Tonetti T, Cressoni M, Cadringher P, Herrmann P, Moerer O, et al. Ventilator-related causes of lung injury: the mechanical power. Intensive Care Med. 2016;42(10):1567-75.

5. Otis AB, Fenn WO, Rahn H. Mechanics of breathing in man. J Appl Physiol. 1950;2(11):592-607.

6. Marhong JD, Telesnicki T, Munshi L, Del Sorbo L, Detsky M, Fan E. Mechanical ventilation during extracorporeal membrane oxygenation. An international survey. Ann Am Thorac Soc. 2014;11(6):956-61.

7. Peek GJ, Mugford M, Tiruvoipati R, Wilson A, Allen E, Thalanany MM, Hibbert CL, Truesdale A, Clemens F, Cooper N, Firmin RK, Elbourne D; CESAR trial collaboration. Efficacy and economic assessment of conventional ventilatory support versus extracorporeal membrane oxygenation for severe adult respiratory failure (CESAR): a multicentre randomised controlled trial. Lancet. 2009;374(9698):1351-63. Erratum in Lancet. 2009;374(9698):1330.

8. Schmidt M, Stewart C, Bailey M, Nieszkowska A, Kelly J, Murphy L, et al. Mechanical ventilation management during extracorporeal membrane oxygenation for acute respiratory distress syndrome: a retrospective international multicenter study. Crit Care Med. 2015;43(3):654-64.

9. Schmidt M, Pellegrino V, Combes A, Scheinkestel C, Cooper DJ, Hodgson C. Mechanical ventilation during extracorporeal membrane oxygenation. Crit Care. 2014;18(1):203.

10. Park M, Azevedo LC, Mendes PV, Carvalho CR, Amato MB, Schettino GP, et al. First-year experience of a Brazilian tertiary medical center in supporting severely ill patients using extracorporeal membrane oxygenation. Clinics (Sao

Paulo). 2012;67(10):1157-63.

11. Serpa Neto A, Schmidt M, Azevedo LC, Bein T, Brochard L, Beutel G, Combes A, Costa EL, Hodgson C, Lindskov C, Lubnow M, Lueck C, Michaels AJ, Paiva JA, Park M, Pesenti A, Pham T, Quintel M, Marco Ranieri V, Ried M, Roncon-Albuquerque R Jr, Slutsky AS, Takeda S, Terragni PP, Vejen M, Weber-Carstens S, Welte T, Gama de Abreu M, Pelosi P, Schultz MJ; ReVA Research Network and the PROVE Network Investigators. Associations between ventilator settings during extracorporeal membrane oxygenation for refractory hypoxemia and outcome in patients with acute respiratory distress syndrome: a pooled individual patient data analysis: mechanical ventilation during ECMO. Intensive Care Med. 2016;42(11):1672-84.

12. Broman M, Frenckner B, Bjällmark A, Broomé M. Recirculation during veno-venous extra-corporeal membrane oxygenation--a simulation study. Int J Artif Organs. 2015;38(1):23-30.

13. Abrams D, Bacchetta M, Brodie D. Recirculation in venovenous extracorporeal membrane oxygenation. ASAIO J. 2015;61(2):115-21.

14. Sreenan C, Osiovich H, Cheung PY, Lemke RP. Quantification of recirculation by thermodilution during venovenous extracorporeal membrane oxygenation. J Pediatr Surg. 2000;35(10):1411-4.

15. Park M, Costa EL, Maciel AT, Silva DP, Friedrich N, Barbosa EV, et al. Determinants of oxygen and carbon dioxide transfer during extracorporeal membrane oxygenation in an experimental model of multiple organ dysfunction syndrome. PLoS One. 2013;8(1):e54954.

16. Nunes LB, Mendes PV, Hirota AS, Barbosa EV, Maciel AT, Schettino GP, Costa EL, Azevedo LC, Park M; ECMO Group. Severe hypoxemia during veno-venous extracorporeal membrane oxygenation: exploring the limits of extracorporeal respiratory support. Clinics (Sao Paulo). 2014;69(3):173-8.

17. Douglas AR, Jones NL, Reed JW. Calculation of whole blood CO_2 content. J Appl Physiol (1985). 1988;65(1):473-7.

18. Mallat J, Mohammad U, Lemyze M, Meddour M, Jonard M, Pepy F, et al. Acute hyperventilation increases the central venous-to-arterial PCO_2 difference in stable septic shock patients. Ann Intensive Care. 2017;7(1):31.

19. Lumb AB, Slinger P. Hypoxic pulmonary vasoconstriction: physiology and anesthetic implications. Anesthesiology. 2015;122(4):932-46.

4. APENDIX

The following scripts were written in C language in R free source software.

4.1. Script for $SatO_2$ looping

```
require(Hmisc)

# Variables
VO2 <- 200
Hb <- 10
CO <- 5
PaCO2 <- 40
FiO2v <- 30
BF <- 3500
Pshunt <- 90
SpO2 <- 64

# Looping preparation
SatO2 <- SpO2
SatO2 <- as.vector(SatO2)
o2t1<- 0

#### Recirculation term
BF <- BF - (0.291321*(1-0.2791^(BF/(1000*CO))) * BF)

# 20 Iteractions
for (i in 1:20){

#### Peripheral compartment
   SinO2 <- SpO2/100 - (VO2/(CO*10*Hb*1.36))
   Cin <- SinO2*1.36*Hb

#### ECMO oxygenation
   PO2post <- 130.5 + 319.5 * exp(1) ^ (-0.0005 * BF)
   Cpost <- 0.0031*PO2post + Hb*1.36

#### Venous admixture
   Cva <- ((Cpost * BF) + (Cin * (CO*1000-BF)))/(CO*1000)

#### Pulmonary residual oxygenation
   PAO2 <- 653*(FiO2v/100) - (PaCO2/0.8)
   CcO2 <- 0.0031 * PAO2 + 1.36 * Hb
   CaO2 <- ((Pshunt/100) * Cva + (1-(Pshunt/100)) * CcO2)
   SatO2[i] <- SpO2
   SpO2 <- 100*CaO2/(1.36*Hb)

 #### O2 transfer
   o2t1[i] <- (Cpost-Cin)*BF/100
   }

## Plot
windows(20,20)
par(mfrow=c(2,2))
plot(1:i,SatO2,xlim=c(0,20),ylim=c(0,100),axes=T,at=-1000,
```

```
    xlab="Number of iteraction (Cycle)",
    ylab=expression(paste("SatO"[2]," (%)")),
    pch=19,type="o",cex=1)
axis(1,at=seq(0,i,1),labels=F)
axis(2,at=seq(0,100,10),las=2)
minor.tick(10,20,0.5)
text(seq(0,i,1), par("usr")[3]-5,
    labels = seq(0,i,1),
    srt = 45,
    xpd = TRUE, cex=1)
abline(v=seq(0,i,1),col="lightgray",lty=2)
abline(h=seq(0,100,10),col="lightgray",lty=2)
```

4.2. Script for P_aCO_2 looping

```
library(Hmisc)

#### Variables
co <- 8
vco2 <- 180
hb <- 10
ph <- 7.40
temp <- 37
BF <- 3500
sweep <- 3.5


Pshunt <- 95
etco2 <- 2
vt <- 10
RR <- 10

co2 <- 62
co2 <- as.vector(co2)

## first loop
for (l in 1:24){
BF <- 250 * l

# Recirculation term and constants
BF <- BF - (0.291321*(1-0.2791^(BF/(1000*co))) * BF)
cco2ad <- vco2/(co*10)
pk <- 6.086+(0.042*(7.4-ph))+((38-temp)*0.00472+(0.00139*(7.4-ph)))
s <- 0.0307+(0.00057*(37-temp))+(0.00002*(37-temp)^2)
sato2 <- 64
co2a <- 62

## Loop start
for (i in 1:800){

  # cco2v calculus
  plasmaco2 <- 2.226*s*co2a*(1+10^(ph-pk))
  cco2a <- (1-((0.0289*hb)/(3.352-0.456*(sato2/100)*(8.142-ph))))*plasmaco2
  cco2v <- cco2a + cco2ad
  plasmaco2v <- cco2v/((1-((0.0289*hb)/(3.352-0.456*(sato2/100)*(8.122-ph)))))
  co2v <- plasmaco2v/(2.226*s*(1+10^(ph-pk-0.02)))
```

```
  # ECMO co2 transfer
  co2t <- (BF*0.017)+(sweep*32.91)+(co2v*1.192)-(hb*3.478)
  ecmocco2ret <- (BF * cco2v / 100) - co2t
  ccpostco2 <- ecmocco2ret*100/BF
  # Venous admixture
  cco2va <- (BF/(co*1000)*ccpostco2) + (((co*1000)-BF)/(co*1000)*cco2v)

  # Lung ventilation & decarboxilation
  mvco2 <- RR*vt*etco2/690
  Pshunt <- Pshunt+0.1
  cdcco2 <- (cco2va*(co*1000*(1-(Pshunt/100)))/100)-mvco2
  cco2plung <- cdcco2*100/(co*1000*(1-(Pshunt/100)))
  cco2a <- cco2va*(Pshunt/100) + cco2plung*(1-(Pshunt/100))

  ## CO2a calculus & Loop end
  plasmaco2a <- cco2a/((1-((0.0289*hb)/(3.352-0.456*(sato2/100)*(8.142-ph)))))
  co2a <- plasmaco2a/(2.226*s*(1+10^(ph-pk)))
}
co2[l] <- co2a
}

## Plot
windows(20,10)
par(mfrow=c(1,2))
plot(-10,-10,xlim=c(0,24),ylim=c(0,100),axes=T,at=-1000,
   xlab=expression(paste("Q"[ECMO]," (mL / minute)")),
   ylab=expression(paste("P"[a],"CO"[2]," (mmHg)")),
   pch=19,type="o",cex=0.5)
axis(1,at=seq(0,l,1),labels=F)
axis(2,at=seq(0,100,10),las=2)
minor.tick(10,10,0.5)
text(seq(0,l,1)-0.2, par("usr")[3]-7,
   labels = seq(0,6000,250),
   srt = 45,
   xpd = TRUE, cex=0.8)
abline(v=seq(0,l,1),col="lightgray",lty=2)
abline(h=seq(0,100,5),col="lightgray",lty=2)
points(1:l,co2,pch=19,type="o",cex=1)
```
